# Endogenous reactive oxygen species cause astrocyte defects and neuronal dysfunctions in the hippocampus: a new model for aging brain

**DOI:** 10.1111/acel.12523

**Published:** 2016-09-13

**Authors:** Takamasa Ishii, Yumi Takanashi, Koichi Sugita, Masaki Miyazawa, Rintaro Yanagihara, Kayo Yasuda, Hiromi Onouchi, Noboru Kawabe, Munehiro Nakata, Yorihiro Yamamoto, Phil S. Hartman, Naoaki Ishii

**Affiliations:** ^1^ Department of Molecular Life ScienceTokai University School of Medicine143 ShimokasuyaIseharaKanagawa259‐1193Japan; ^2^ Institute of Medical SciencesTokai University143 ShimokasuyaIseharaKanagawa259‐1193Japan; ^3^ School of Bioscience and BiotechnologyTokyo University of Technology1404‐1 KatakuramachiHachiojiTokyo192‐0982Japan; ^4^ Support Center for Medical Research and EducationTokai University143 ShimokasuyaIseharaKanagawa259‐1193Japan; ^5^ Department of OphthalmologyTokai University School of Medicine143 ShimokasuyaIseharaKanagawa259‐1193Japan; ^6^ Department of Applied BiochemistryTokai University School of Engineering4‐1‐1 KitakanameHiratsukaKanagawa259‐1292Japan; ^7^ Department of BiologyTexas Christian UniversityFort WorthTX76129USA; ^8^ Present address: Department of Biological SciencesNorth Carolina State UniversityRaleighNC27695USA

**Keywords:** aging, astrocyte, Ca^2+^, JNK/SAPK, mitochondria, oxidative stress

## Abstract

The etiology of astrocyte dysfunction is not well understood even though neuronal defects have been extensively studied in a variety of neuronal degenerative diseases. Astrocyte defects could be triggered by the oxidative stress that occurs during physiological aging. Here, we provide evidence that intracellular or mitochondrial reactive oxygen species (ROS) at physiological levels can cause hippocampal (neuronal) dysfunctions. Specifically, we demonstrate that astrocyte defects occur in the hippocampal area of middle‐aged *Tet‐mev‐1* mice with the SDHC^V69E^ mutation. These mice are characterized by chronic oxidative stress. Even though both young adult and middle‐aged *Tet‐mev‐1* mice overproduced MitoSOX Red‐detectable mitochondrial ROS compared to age‐matched wild‐type C57BL/6J mice, only young adult *Tet‐mev‐1* mice upregulated manganese and copper/zinc superoxide dismutase (Mn‐ and Cu/Zn‐SODs) activities to eliminate the MitoSOX Red‐detectable mitochondrial ROS. In contrast, middle‐aged *Tet‐mev‐1* mice accumulated both MitoSOX Red‐detectable mitochondrial ROS and CM‐H_2_DCFDA‐detectable intracellular ROS. These ROS levels appeared to be in the physiological range as shown by normal thiol and glutathione disulfide/glutathione concentrations in both young adult and middle‐aged *Tet‐mev‐1* mice relative to age‐matched wild‐type C57BL/6J mice. Furthermore, only middle‐aged *Tet‐mev‐1* mice showed JNK/SAPK activation and Ca^2+^ overload, particularly in astrocytes. This led to decreasing levels of glial fibrillary acidic protein and S100β in the hippocampal area. Significantly, there were no pathological features such as apoptosis, amyloidosis, and lactic acidosis in neurons and astrocytes. Our findings suggest that the age‐dependent physiologically relevant chronic oxidative stress caused astrocyte defects in mice with impaired mitochondrial electron transport chain functionality.

## Introduction

It has recently been reported that astrocyte defects such as astrogliosis, astroglial death and astrocytic beading (clasmatodendrosis) are pathophysiological phenotypes in neuronal degenerations, frontotemporal dementia, ischemic brain injury or status epilepticus. However, the causes and effects of these phenomena remain incompletely understood. In 1961, Friede and Houten demonstrated that the experimental incubation of cerebellar tissue from rats in glucose or in glucose‐6‐phosphate in the presence of cyanide resulted in clasmatodendrosis of astrocytes and marked edematous changes, particularly in the neuroglia of the Purkinje layer (Friede *et al*. 1961). In addition, Hulse *et al*. (2001) demonstrated astrocytic beading (clasmatodendrosis) in hippocampal organ cultures. Astrocytes in the periventricular white matter exhibited clasmatodendrosis, defined as cytoplasmic swelling and vacuolation of astroglia, with disintegrating and beading of their dendrites in Alzheimer's disease (AD) and cerebrovascular disease patients (Tomimoto *et al*., 1997; Sahlas *et al*., 2002). Other reports demonstrated that astrocytes showed enlarged, irregular shapes and vacuolation. In addition, their processes appeared fragmented in frontotemporal dementia (FTD; Su *et al*., 2000; Martin *et al*., 2001). Furthermore, astrocytic clasmatodendrosis and death were caused after hypoxic–ischemic injury in developing brains of rats and humans (Gelot *et al*., 2009). Ischemia and reperfusion cause acute oxidative stress and result in necrotic cell death and severe injury. It has also been reported that astrocytic clasmatodendrosis is closely associated with disseminated selective neuronal cell death and maturation of injury after ischemia. The thinned out astrocytic end‐feet with degenerated mitochondria, which are represented as degeneration of astrocytic processes (APs), is exhibited in neuropil (Ito *et al*., 2009). We hypothesized that these astrocyte defects could be also by induced by chronic oxidative stress.

We have previously investigated the short‐lived *mev‐1* mutant of the nematode *Caenorhabditis elegans*, which was isolated based upon its hypersensitivity to the reactive oxygen species (ROS)‐generating chemical *me*thyl *v*iologen (Ishii *et al*., 1990). In addition to its hypersensitivity to oxidative stress, *mev‐1* mutants aged precociously under hyperoxia (Honda *et al*., 1993; Hosokawa *et al*., 1994; Adachi *et al*., 1998). The *mev‐1*(*kn‐1*) mutation, which resulted in an amino acid substitution at the 71st position from glycine to glutamate (G71E), was identified as residing in the putative gene *cyt‐1* [succinate dehydrogenase (SDH) cytochrome *b* large subunit in complex II, a human *SDHC* gene homologue] (Ishii *et al*., 1998). These mutants showed elevated mitochondrial ROS that was caused by the compromised complex II leading to electron leakage from the electron transport system (Senoo‐Matsuda *et al*., 2001).

Recently, we established *mev‐1*‐mimic conditional transgenic mice (*Tet‐mev‐1*) with the equivalent mutation in SDHC using our modified Tet On/Off tetracycline system. Expression was uniquely controlled by both rTetR (recombinant tetracycline receptor)‐VP16 (reverse tetracycline‐dependent transcriptional activator) and TetR‐KRAB (tetracycline‐dependent transcriptional repressor; Ishii *et al*., 2011). The *mev‐1*‐mimic mutation results in the substitution of valine for glutamic acid at position 69 (V69E; Ishii *et al*., 2005; Ishii *et al*., 2013). The *Tet‐mev‐1* mice ubiquitously and competitively expressed the *mev‐1*‐mimic SDHC^V69E^ mutation coding transgene at levels less than the endogenous *SDHC* gene in various tissues. It is important to note that this model elevated oxidative stress caused by the electron leakage from genetically impaired mitochondrial electron transport system in some predicted tissues with complex II activity, even though this was not a tissue‐specific conditional transgenic animal model. *Tet‐mev‐1* conditional transgenic mice grew to normal size in 12 weeks after suffering of low birthweight and initial growth retardation. They had low fertility and recurrent miscarriages (Ishii *et al*., 2011, 2014). After normal development, male and female *Tet‐mev‐1* mice showed accelerated corneal dysfunctions with age, *that is*, delayed epithelialization with keratitis, decreasing endothelial cells such as Fuch's corneal dystrophy, and thickened Descemet's membrane (Onouchi *et al*., 2012). Also, male *Tet‐mev‐1* mice developed lacrimal gland inflammation resulting in dry eyes (Uchino *et al*., 2012). A number of pathophysiological phenotypes attributable to oxidative stress [*e.g.,* elevated carbonylated protein and 8‐oxoguanine (8‐OHdG) levels] were significantly higher in 12‐month‐old *Tet‐mev‐1* mice compared to age‐matched wild‐type C57BL/6J mice.

In this report, we assessed the effects of age‐dependent oxidative stress in the hippocampus induced by genetically impaired mitochondrial electron transport. We also explored whether this physiologically relevant chronic oxidative stress could lead to age‐dependent brain dysfunction and astrocyte defects in mice.

## Results

### *mev‐1‐*mimic mutated SDHC^V69E^‐inducible *Tet‐mev‐1* mice

We first measured wild‐type and mutant SDHC protein levels in the hippocampal area. The SDHC protein level, including the SDHC^V69E^ protein, was increased 1.7 times in doxycycline‐treated *Tet‐mev‐1* mice compared to wild‐type C57BL/6J mice under the same conditions. Rather than using *Tet‐mev‐1* mice that were not treated with doxycycline, C57BL/6J mice with doxycycline treatment served as controls in all experiments. This eliminated the possibility of abnormal levels of oxidative stress in the control mice, which might have occurred in the *Tet‐mev‐1* mice even when SDHC^V69E^ was not induced. The SDHC level was experimentally identical in doxycycline‐treated and untreated C57BL/6J mice and untreated *Tet‐mev‐1* mice. We also determined the ROS levels in submitochondrial particles of the hippocampal area in the *mev‐1*‐mimic mutant SDHC^V69E^ *Tet‐mev‐1* and wild‐type C57BL/6J mice. As expected, the ROS level was elevated by 1.5‐fold compared to that of wild‐type C57BL/6J mice as measured using a previously reported *in vitro* assay (Fig. S1A,B; Ishii *et al*., 2011).

Young adult (4–8 months old), middle‐aged (10–14 months old), and senior‐aged (24–28 months old) *Tet‐mev‐1* and wild‐type C57BL/6J mice were then examined under the doxycycline treatment. They were found to be equivalent in terms of β‐amyloid 1–42 accumulation in astrocytes by immunohistochemistry using β‐amyloid 1–42 antibody. There was no pathological abnormality such as amyloidosis in any age‐group (Fig. S2). As well, there was no detectable apoptotic cell death as measured by TUNEL staining in the hippocampal area of any of the age‐grouped mice (Fig. S3). In addition, the lactate accumulation level, which can be increased in rare neurodegenerative disorders such as mitochondrial myopathy, encephalopathy, lactic acidosis, and stroke‐like episodes syndrome (Salsano *et al*., 2011; Emmanuele *et al*., 2012), was not different in the hippocampal area of *Tet‐mev‐1* mice as compared to the age‐matched wild‐type C57BL/6J mice at each age (Fig. S4). These results indicate that there were no detectable pathological features (*e.g.,* β‐amyloid 1–42 amyloidosis, apoptosis, and lactic acidosis) in the brain of young adult, middle‐aged, and senior‐aged *Tet‐mev‐1* mice. This strongly suggests that the abnormalities we document below are not secondary consequences of, for example, lactic acidosis.

### Mitochondrial and intracellular ROS levels with age

**Figure 1:**
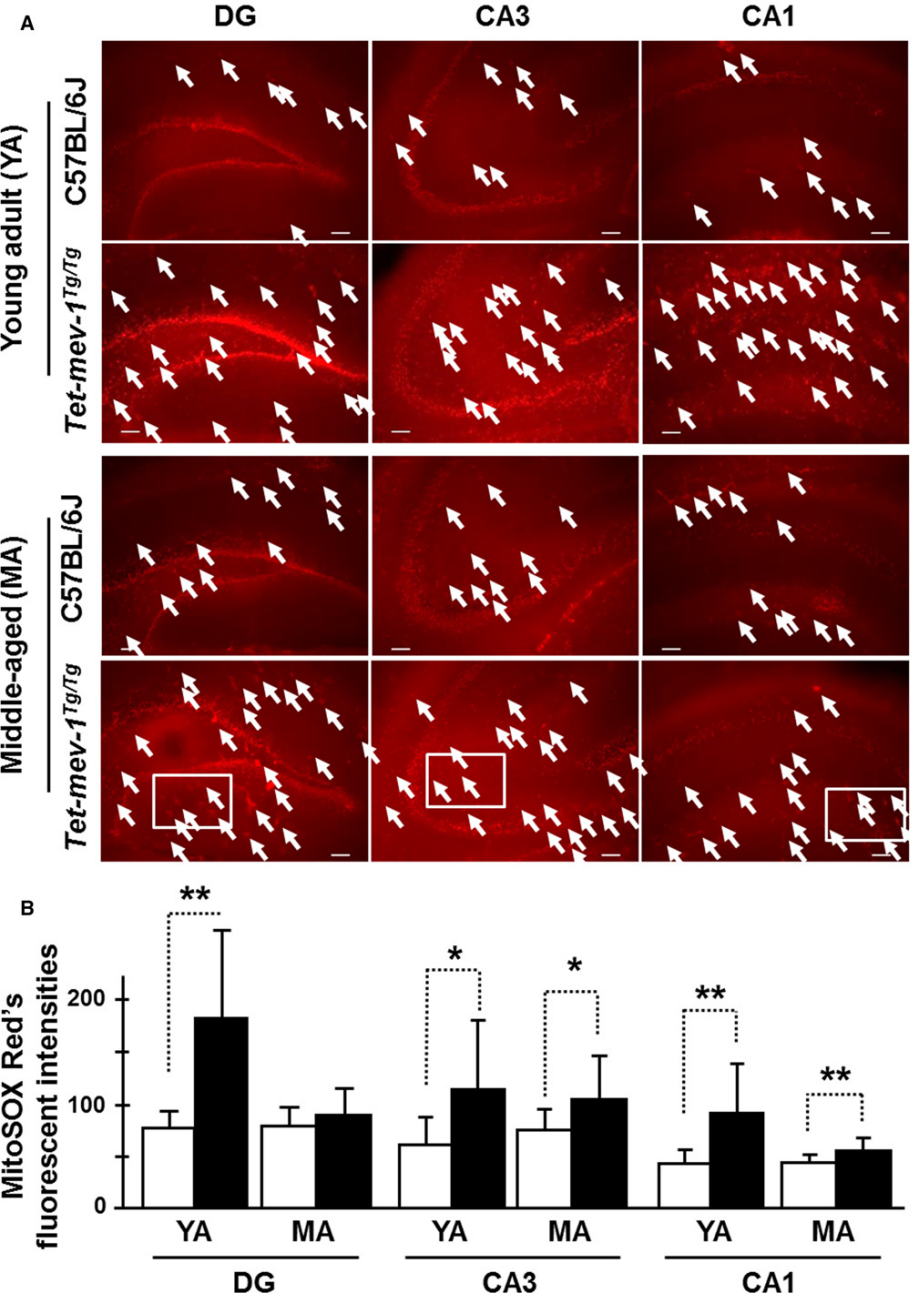
Mitochondrial reactive oxygen species (ROS) production levels. (A) Micrographs of mitochondrial ROS‐generated red fluorescence detected by the MitoSOX Red chemical probe on *in vitro*‐cultured hippocampal tissue sections. The *in vitro*‐cultured hippocampal tissue sections were treated with HQNO (a mitochondrial electron transport chain inhibitor) and were measured for confirmation of *in vitro* production level. White arrows indicate the fluorescent‐positive astrocytes. Scale bar = 100 μm. White squares are described with higher magnification images in Fig. S5A (Supporting information). (B) The statistical bar charts of red fluorescent intensities on the dentate gyrus (DG), CA3, and CA1 regions of young adult (YA) and middle‐aged (MA) mice, respectively. The white and black bars indicate the MitoSOX Red's fluorescent intensities in wild‐type C57BL/6J and *Tet‐mev‐1* mice, respectively. Data are expressed as mean ± SD; *P <* *0.05; **P <* *0.01; *n *=* *3 in each group.

**Figure 2:**
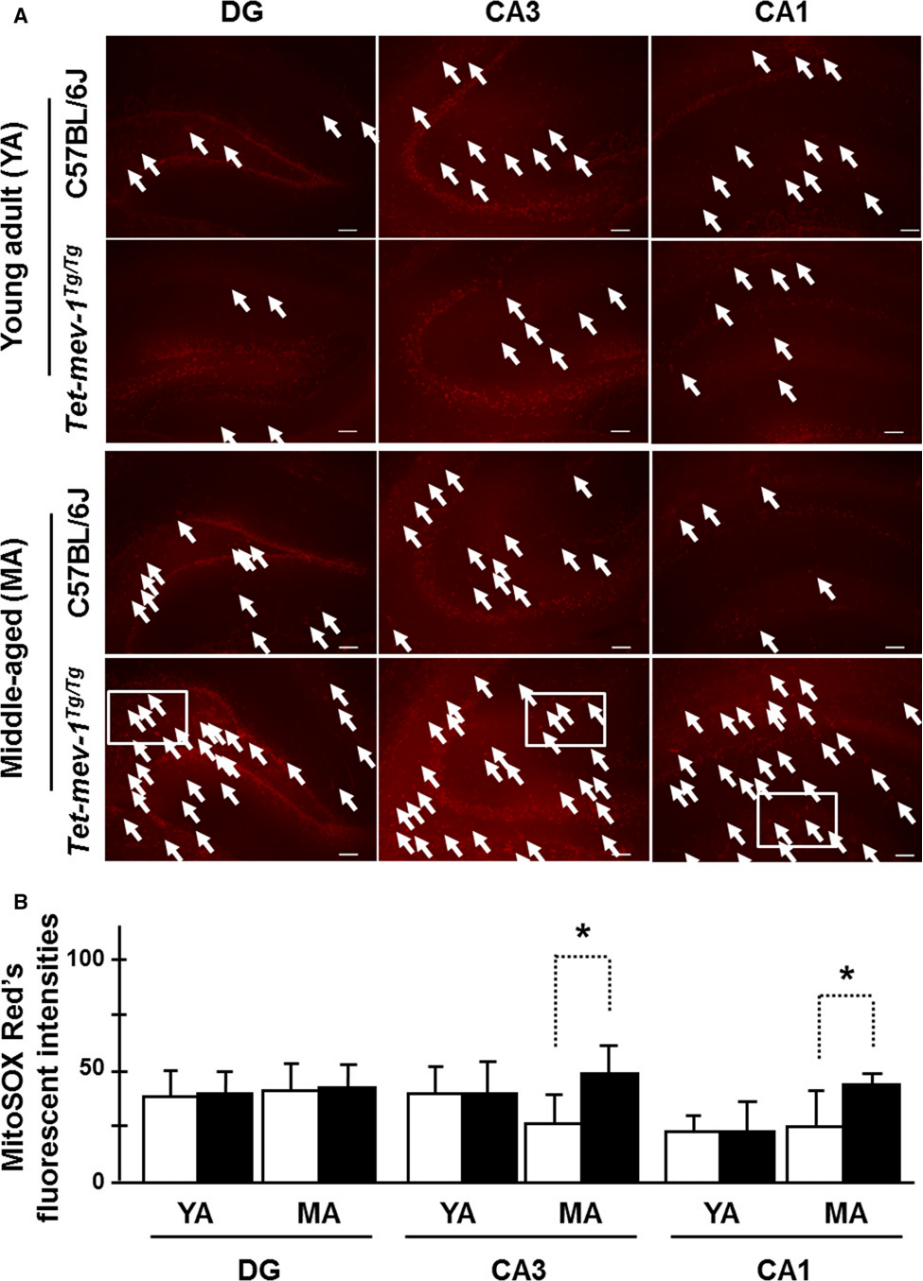
Mitochondrial reactive oxygen species (ROS) accumulation levels. (A) Micrographs of mitochondrial ROS‐generated fluorescence detected by the MitoSOX Red chemical probe on *in vitro*‐cultured hippocampal tissue sections for confirmation of *in vivo* accumulation levels. White arrows indicate the fluorescent‐positive astrocytes. Scale bar = 100 μm. White squares are described with higher magnification images in Fig. S5B (Supporting information). (B) The statistical bar charts of red fluorescent intensities on the dentate gyrus (DG), CA3, and CA1 regions of young adult (YA) and middle‐aged (MA) mice, respectively. The white and black bars indicate the MitoSOX Red's fluorescent intensities in wild‐type C57BL/6J and *Tet‐mev‐1* mice, respectively. Data are expressed as mean ± SD; *P <* *0.05; *n *=* *3 in each group.

Mitochondrial ROS levels were measured using MitoSOX Red on *in vitro*‐cultured tissue sections of the hippocampal area in the presence or absence of the semiquinone‐like inhibitor 2‐*n*‐heptyl‐4‐hydroxyquinoline‐*N*‐oxide (HQNO) for the measurement of production or accumulation levels of ROS, respectively. Mitochondrial ROS, mainly superoxide which was confirmed by MitoSOX Red fluorescence, was generated at particularly high levels in most of the neurons in granular and pyramidal layers (Figs 1A and 2A). Mitochondrial ROS production levels *in vitro* with artificial (HQNO‐induced) ROS production were increased in the granule and pyramidal cells including cells of other areas present in the HQNO‐treated hippocampal sections of young adult and middle‐aged *Tet‐mev‐1* mice compared to those of the age‐matched wild‐type C57BL/6J mice (Fig. 1A). From a statistical perspective, production levels were dramatically decreased in middle‐aged *Tet‐mev‐1* mice relative to young adult *Tet‐mev‐1* mice, excepting in the CA3 region (Fig. 1B). This result indicates that the mitochondrial ROS production was higher under conditions of cell proliferation and differentiation and was downregulated in middle‐aged *Tet‐mev‐1* mice, especially the DG region.

However, somewhat surprisingly, there was no statistical difference in MitoSOX Red fluorescent intensities in the granule and pyramidal cells of young adult *Tet‐mev‐1* vs. wild‐type C57BL/6J mice in the absence of HQNO, indicating equivalent mitochondrial ROS accumulation levels *in vivo* under the physiologically maintained SOD activity (Fig. 2A,B). On the other hand, the middle‐aged *Tet‐mev‐1* mice had statistically elevated accumulation levels in cells of pyramidal layer and the other areas of the CA3 and CA1 regions (Fig. 2A,B). In the DG region of middle‐aged *Tet‐mev‐1* mice, the accumulation level was not statistically elevated in granule cells, but there was frequently more detected in cells of the other areas (Fig. 2B). There are strongly fluorescent cells, and it seems to astrocytes with large cell body in the other areas (Fig. S5A,B).

**Figure 3:**
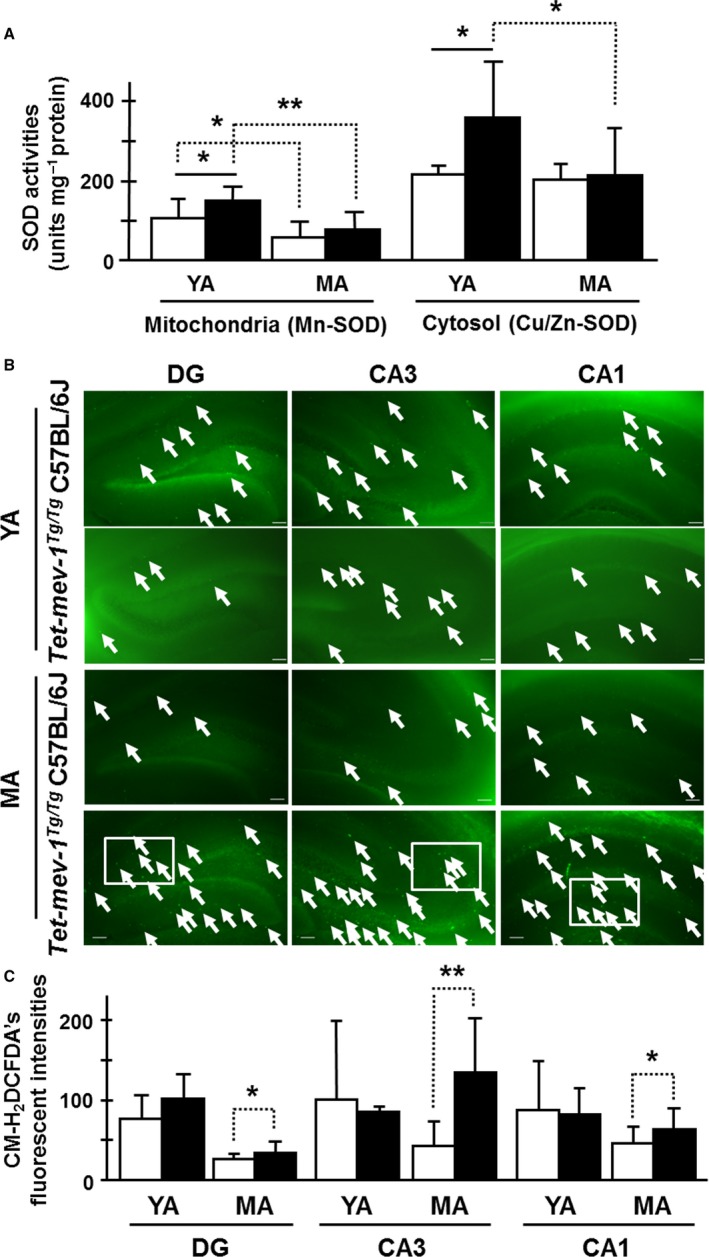
Superoxide dismutase (SOD) activities and intracellular ROS levels. (A) SOD activities in mitochondrial (Mn‐SOD) and cytosolic (Cu/Zn‐SOD) protein fractions of the hippocampal area. The white and black bars indicate the SOD activities (units per mg protein) in wild‐type C57BL/6J and *Tet‐mev‐1* mice, respectively. Data are expressed as mean ± SD; *P <* *0.05; **P <* *0.01; *n *=* *7 in each group. (B) Micrographs of intracellular ROS‐generated green fluorescence by CM‐H_2_DCFDA on *in vitro*‐cultured hippocampal tissue sections. White arrows indicate fluorescent‐positive astrocytes. Scale bar = 100 μm. White squares are described with higher magnification images in supplemental information, Fig. S5C. (C) The statistical bar charts of green fluorescent intensities on the dentate gyrus (DG), CA3, and CA1 regions of young adult (YA) and middle‐aged (MA) mice, respectively. The white and black bars indicate the CM‐H_2_DCFDA's fluorescent intensities in wild‐type C57BL/6J and *Tet‐mev‐1* mice, respectively. Data are expressed as mean ± SD; *P <* *0.05; **P <* *0.01; *n *=* *3 in each group.

We then compared the ability to scavenge ROS in *Tet‐mev‐1* and wild‐type C57BL/6J mice at each age. We first measured manganese and copper/zinc superoxide dismutase (Mn‐ and Cu/Zn‐SOD) activities in *Tet‐mev‐1* and wild‐type C57BL/6J mice. Both Mn‐ and Cu/Zn‐SOD activities were approximately 1.5‐fold higher in young adult *Tet‐mev‐1* mice than in the age‐matched wild‐type C57BL/6J mice (Fig. 3A). However, the Mn‐SOD activity in middle‐aged *Tet‐mev‐1* and wild‐type C57BL/6J mice was approximately equal, decreasing to roughly half from that in each young adult (Fig. 3A). In contrast, Cu/Zn‐SOD activity was not changed in wild‐type mice with age, but significantly decreased in middle‐aged *Tet‐mev‐1* mice compared to that of young adult *Tet‐mev‐1* mice (Fig. 3A).

Next, total intracellular ROS levels were determined using 5‐(and‐6)‐chloromethyl‐2′,7′‐dichlorodihydrofluorescein diacetate, acetyl ester (CM‐H_2_DCFDA) on *in vitro*‐cultured tissue sections of the hippocampal area. Interestingly, the CM‐H_2_DCFDA chemical fluorescence, which detects primarily hydrogen peroxide and hydroxyl radicals, was particularly strong in cells of the other areas, not granular and pyramidal layers, even though those shapes are like particle aggregates different from MitoSOX Red's fluorescence (Figs 3B and S5C). The levels were statistically increased in middle‐aged *Tet‐mev‐1* mice compared to the age‐matched wild‐type C57BL/6J mice, even though levels were lower than in young adult mice (Fig. 3C). These CM‐H_2_DCFDA‐detectable intracellular ROS levels were similar to the accumulation levels of MitoSOX Red‐detectable mitochondrial ROS dependent on mitochondrial electron transport activation. This result suggests that oxidative stress was induced in a physiological range by the impaired mitochondria of middle‐aged *Tet‐mev‐1* mice. Next, we analyzed the redox stability, and thiol and glutathione (GSH) levels to confirm that oxidative stress was in a physiological range.

### Redox stability, redox signal transduction activity, and Ca^2+^ influx

Thiols are effective nonenzymatic or enzymatic scavengers of hydrogen peroxide. Consequently, decreased thiol levels, particularly GSH, are an oxidative stress indicator (Chinta *et al*., 2007; Danielson *et al*., 2011). Total thiols and GSH levels increased in both animals with age, but there were no differences between *Tet‐mev‐1* and wild‐type C57BL/6J mice (Fig. S6A,B). Therefore, although glutathione disulfide (GSSG) levels were increased in middle‐aged *Tet‐mev‐1* and wild‐type C57BL/6J mice compared to young adults, the GSSG/GSH ratio of *Tet‐mev‐1* mice was the same as wild‐type C57BL/6J mice in both ages (Fig. S6B,C). This result suggests that intracellular ROS, especially hydrogen peroxide, were effectively scavenged by nonenzymatic or enzymatic antioxidant abilities under these physiologically homeostatic conditions. Consequently, carbonylated protein ratios, an indicator of the oxidative stress on intracellular components, were normal for both young adult and middle‐aged *Tet‐mev‐1* mice compared to wild‐type C57BL/6J mice in each age (Fig. S7). In summary, these results indicate that despite more ROS generation in *Tet‐mev‐1* mice, the resultant oxidative stress was controlled to physiologically normal levels in both aged *Tet‐mev‐1* mice.

**Figure 4:**
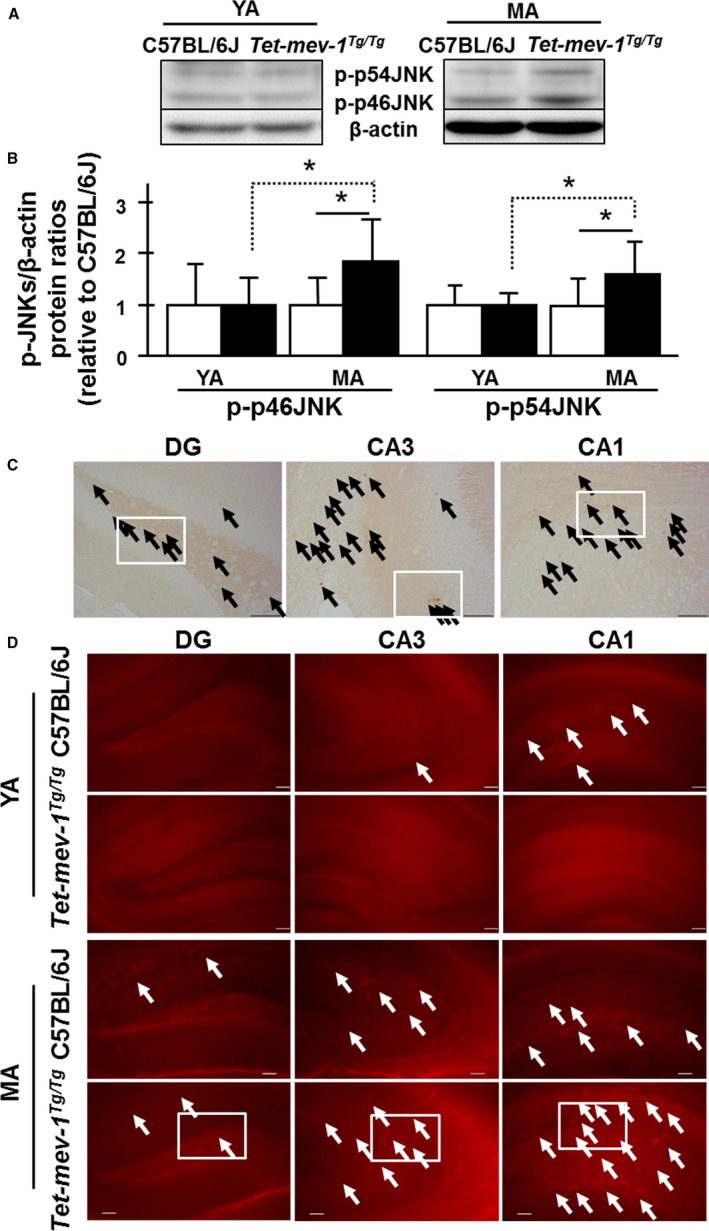
c‐Jun N‐terminal kinases (JNK)/stress‐activated protein kinase (SAPK)‐activated and Ca^2+^‐overloaded cells. (A) Western blot images using phospho‐JNK/SAPK (Thr183/Tyr185) and β‐actin antibodies to total protein lysate in hippocampal area. (B) The statistically internal standardized ratios of phosphorylated p46‐ and p54‐JNK/SAPKs (JNK1 and 2) by β‐actin level relative to young adult wild‐type C57BL/6J. White and black bars indicate the wild‐type C57BL/6J and *Tet‐mev‐1* mice, respectively. Data are expressed as mean ± SD; *P <* *0.05; *n *=* *4 in each group. (C) Micrographs of immunohistochemical analysis using phospho‐SAPK/JNK (Thr183/Tyr185) antibody. Black arrows indicate the immunostaining positive brown cells. White squares are described with higher magnification images in Fig. S8A (Supporting information). (D) Micrographs of Ca^2+^‐overloaded red fluorescent cells with Rhod‐2‐AM. White arrows indicate Ca^2+^‐overloaded red fluorescent‐positive astrocytes. Scale bar = 100 μm. White squares are described with higher magnification images in Fig. S8B (Supporting information).

We next looked in the hippocampal areas at levels of c‐Jun N‐terminal kinases/stress‐activated protein kinases (JNK/SAPKs), which control oxidative stress‐responsible signal transduction pathways (Zhu *et al*., 2004). Phosphorylated forms of p46‐ and p54‐JNK/SAPKs (JNK1 and 2) were increased approximately twofold in middle‐aged *Tet‐mev‐1* mice compared to same aged wild‐type C57BL/6J mice (Fig. 4A,B). Specifically, the phosphorylated forms of p46‐ and p54‐JNK/SAPKs (JNK1 and 2) were detected by immunohistochemistry in cells of other areas excepting the granule and pyramidal cells (Figs 4C and S8A). This suggests that the cells seem to astrocytes in middle‐aged *Tet‐mev‐1* mice are subjected to chronic oxidative stress, and it affects intracellular homeostatic adaptations with upregulated redox signal transductions under physiological controls. Additionally, Ca^2+^‐overloaded cells were detected with the rhodamine‐like Ca^2+^ fluorescence indicator Rhod‐2‐AM. Ca^2+^‐overloaded cells were markedly present among the cells of the other areas, not pyramidal layer of middle‐aged *Tet‐mev‐1* mice, especially the CA3 and CA1 regions (Figs 4D and S8B). There are strongly fluorescent cells, and it seems to astrocytes with large cell body in the other layers as MitoSOX Red's fluorescence (Figs S5A,B, and S8B).

### Hippocampal astrocytic condition and neuronal activity as a function of age

**Figure 5:**
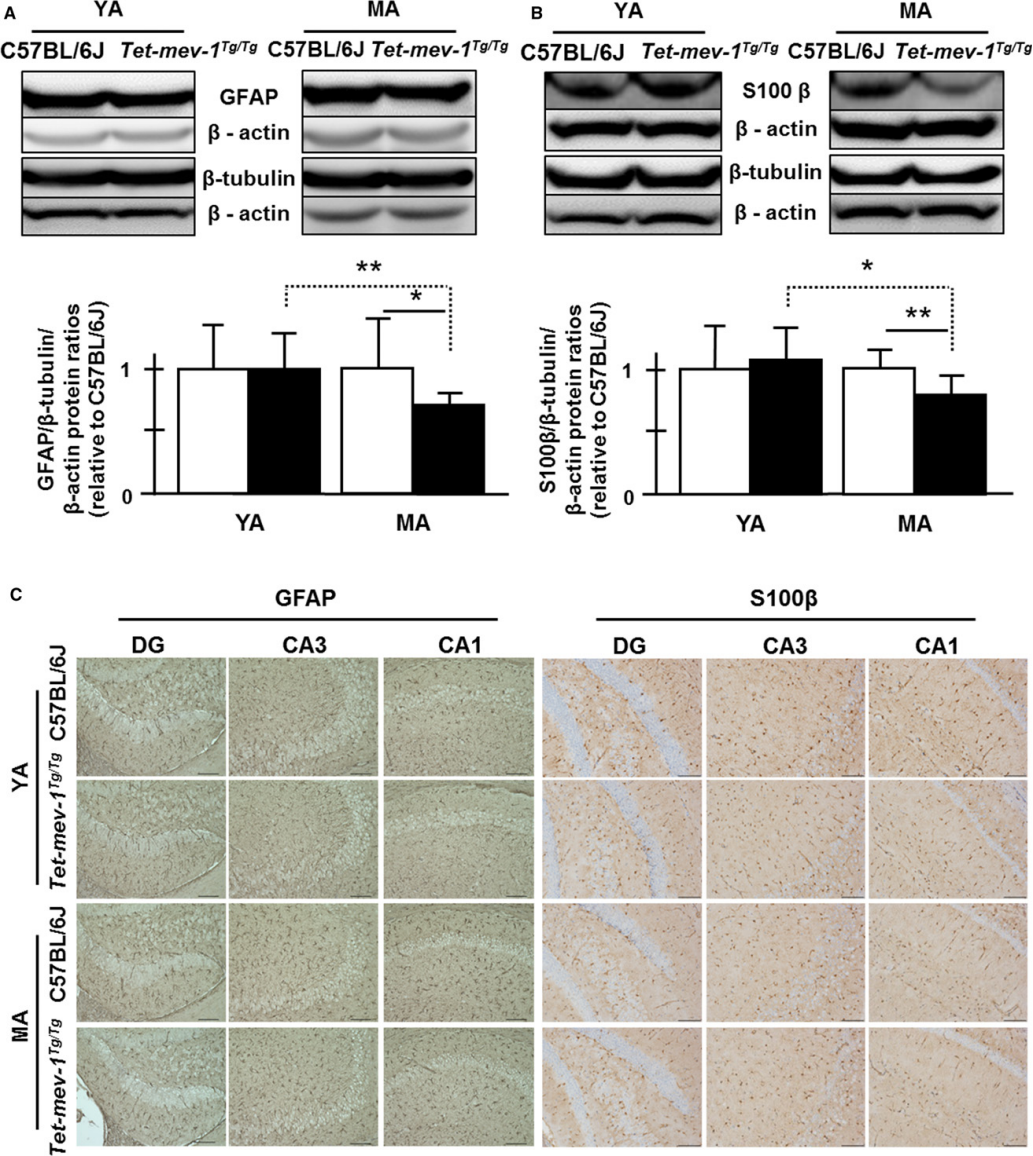
The levels of glial fibrillary acidic protein (GFAP) and S100β as astrocytic protein marker proteins in the hippocampal area. (A) Western blot images using GFAP, tubulin beta‐3, and β‐actin antibodies to total protein lysate in hippocampal area. The statistical results of internal standardized GFAP/β‐tubulin ratio by β‐actin levels relative to young adult wild‐type C57BL/6J. White and black bars indicate the wild‐type C57BL/6J and *Tet‐mev‐1* mice, respectively. Data are expressed as mean ± SD; *P <* *0.05; **P <* *0.01; *n* = >12 in each group. (B) Western blot images using S100β, tubulin beta‐3, and β‐actin antibodies to total protein lysate in hippocampal area. The statistical results of internal standardized S100β/β‐tubulin ratio by β‐actin levels relative to young adult wild‐type C57BL/6J. White and black bars indicate the wild‐type C57BL/6J and *Tet‐mev‐1* mice, respectively. Data are expressed as mean ± SD; *P <* *0.05; **P <* *0.01; *n *= >12 in each group. (C) Micrographs of immunohistochemical analysis on paraffined hippocampal tissue sections using GFAP and S100β antibody. Brown cells indicate GFAP‐stained astrocytes. Scale bar = 100 μm.

We then assessed whether chronic oxidative stress affected neuronal and astrocytic conditions in the hippocampal area. After checking their specificities by immunohistochemistry on the primary cultured cells of *Tet‐mev‐1* mouse brain, tubulin beta‐3 (β‐tubulin), glial fibrillary acidic protein (GFAP), and S100β antibodies were employed to determine the neuronal and astrocytic protein levels by Western blot analysis. The GFAP/β‐tubulin/actin and S100β/β‐tubulin/actin ratios were significantly decreased at middle‐aged *Tet‐mev‐1* mice compared to young adult *Tet‐mev‐1* mice or both ages of wild‐type C57BL/6J mice, even though β‐tubulin levels were not changed (Fig. 5A,B). This agreed with the results of immunohistochemistry using each antibody on the paraffin sections of hippocampal area (Fig. 5C).

**Figure 6:**
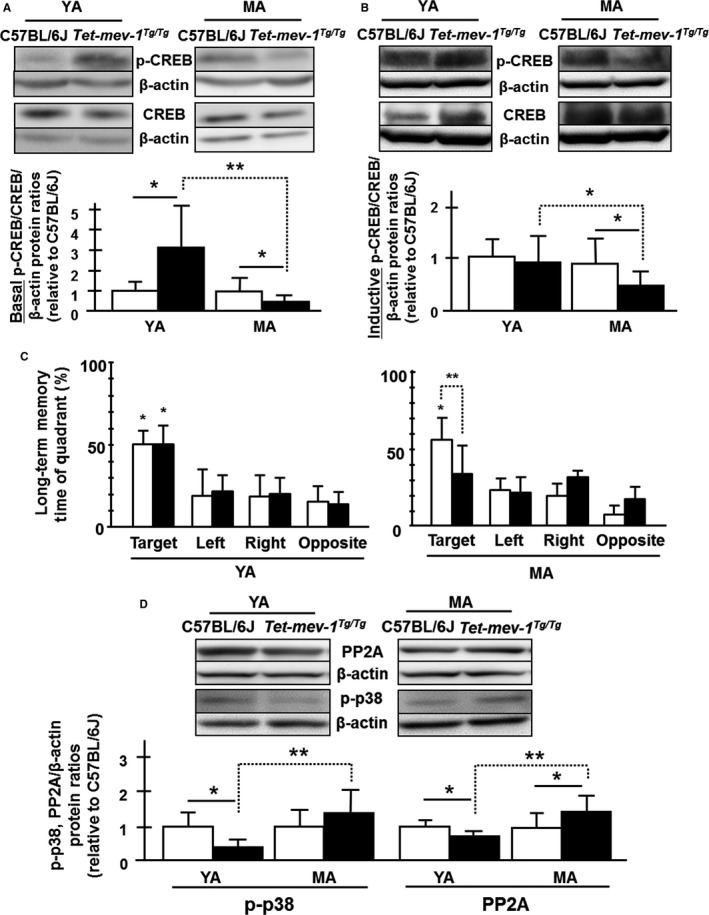
Basal and inductive phosphorylated CREB levels, long‐term spatial memory abilities using Morris water maze, and the upstream key enzyme activities for CREB phosphorylation. (A,B) Western blot images using phospho‐CREB (Ser133) and CREB antibodies to total protein lysate in the hippocampal area. The statistical bar charts of internal standardized basal (A) and inductive (B) phosphorylated CREB/CREB ratios by β‐actin levels relative to young adult wild‐type C57BL/6J. White and black bars indicate the wild‐type C57BL/6J and *Tet‐mev‐1* mice, respectively. All data are expressed as mean ± SD; *P <* *0.05; **P <* *0.01; *n *= >7 in each group (A); *n *=* *6 in each age (B). (C) Long‐term spatial memory test was performed after 5‐day learning using Morris water maze. White and black bars indicate the wild‐type C57BL/6J and *Tet‐mev‐1* mice, respectively. Data are expressed as mean ± SD; *P <* *0.01; **P <* *0.05; *n *= >6 in each group. (D) Western blot images using protein phosphatase 2A (PP2A) C subunit and phospho‐p38 MAP kinase (Thr180/Tyr182) antibodies to total protein lysate in hippocampal area. The statistical bar charts of internal standardized phosphorylated p38 and PP2A C subunit by β‐actin levels relative to young adult wild‐type C57BL/6J. White and black bars indicate the wild‐type C57BL/6J and *Tet‐mev‐1* mice, respectively. All data are expressed as mean ± SD; *P <* *0.05; **P <* *0.01; *n *=* *4 in each group.

Furthermore, neuronal activity in the hippocampal area was evaluated by measuring the activation of CREB (cAMP response element‐binding protein) transcription factor. CREB is phosphorylated to play a key role in neuronal plasticity and long‐term spatial memory. It is important to note that long‐term spatial memory requires new protein syntheses in the hippocampus by the activation of CREB transcriptional factor (Silva *et al*., 1998; Lonze & Ginty, 2002). Basal and inductive phosphorylated CREB/total CREB levels were quantified in Western blot analysis using phosphorylated CREB (Ser133) and CREB antibodies. There was roughly three times more phosphorylated CREB in young adult *Tet‐mev‐1* mice relative to wild‐type C57BL/6J mice (Fig. 6A). In contrast, the ratio dropped from approximately 3.1 to 0.5 in middle‐aged mice, a total reduction of 80% in the two ratios (Fig. 6A). On the other hand, inductive phosphorylated CREB levels that were analyzed immediately after learning using hidden platform trials in a Morris water maze (Figs S9 and S10) were not changed between young adult *Tet‐mev‐1* and C57BL/6J mice (three trials on the first day and two trials on the extraction day, second day; Fig. 6B). However, in middle‐aged *Tet‐mev‐1* mice, the ratio dropped to approximately half as compared to C57BL/6J mice same as basal levels of phosphorylated CREB (Fig. 6A,B). Not surprisingly, hippocampus‐dependent memory consolidation was disrupted in a long‐term memory test using a Morris water maze in middle‐aged *Tet‐mev‐1* mice, reflecting the decreasing of inductive phosphorylated CREB levels. The memory consolidation ability of young adult *Tet‐mev‐1* mice was not changed (Fig. 6C).

We then looked at some upstream regulators of phosphorylated CREB level using Western blot analysis (Bito *et al*., 1996; Silva *et al*., 1998). The levels of phosphorylated‐to‐total protein ratios of PKA (protein kinase A), CaMK (Ca^2+^/CaM‐dependent protein kinase) IV, and PP1 (Ser/Thr protein phosphatase 1) were statistically identical between young adult and middle‐aged *Tet‐mev‐1* mice relative to wild‐type C57BL/6J mice (Fig. S11A–D). As well, the catalytic subunit (calcineurin A) of calcineurin [protein phosphatase 2B (PP2B)] level was also equally expressed in young adult and middle‐aged *Tet‐mev‐1* mice relative to wild‐type C57BL/6J mice (Fig. S11A,E). In contrast, protein phosphatase 2A (PP2A) C subunit and phosphorylated p38 MAPK levels were significantly decreased in young *Tet‐mev‐1* adults relative to wild‐type C57BL/6J mice, and the levels were also significantly increased with age (Fig. 6D). It has previously been reported that PP2A activity, which is required for p38‐mediated dephosphorylation, regulates PKA‐phosphorylated CREB transcriptional activity (Wadzinski *et al*., 1993; Junttila *et al*., 2008; Maalouf & Rho, 2008; Fey *et al*., 2012). Our results are consistent with these past results, which is that the basal phosphorylated CREB level is controlled by the phosphatase activity through p38 MAPK‐PP2A signal transduction.

## Discussion

In this report, we focused on the brain functional changes in response to physiologically relevant chronic oxidative stress produced by genetically impaired mitochondrial respiratory chain activity as a function of age. The changes we detected occurred before mice developed pathological features such as amyloidosis, apoptosis, and lactic acidosis.

First, we confirmed that mitochondrial ROS, mainly superoxide anion, and CM‐H_2_DCFDA‐detectable ROS (mainly hydrogen peroxide and hydroxyl radicals) accumulated at higher levels in most of the granule and pyramidal cells and some cells of other areas, which seem to astrocytes with large cell body, of middle‐aged *Tet‐mev‐1* mice compared to age‐matched wild‐type C57BL/6J mice, even though the levels were lower than young adult mice. The increase in ROS in *Tet‐mev‐1* mice was compensated by the animals’ intracellular antioxidant ability and/or redox signal transduction pathways. As a result, oxidative stress was kept in the physiological range as manifested by identical levels of thiol and disulfide GSH compared to age‐matched wild‐type C57BL/6J mice. Consequently, all ages examined [young adult (4–8 months old), middle‐aged (10–14 months old), and senior‐aged (24–28 months old)] did not develop detectable pathological features such as amyloidosis, apoptosis, and lactic acidosis.

Middle‐aged *Tet‐mev‐1* mice accumulated intracellular ROS particularly in their astrocytes. They displayed diminished glial astrocyte intermediate filament protein (GFAP) and S100β. Those mice with astrocyte defects also showed Ca^2+^ overload and JNK/SAPK activation. JNK/SAPK is a member of the stress‐activated MAPK signal transduction pathway and has been linked to the cell death and redox signaling, so‐called cell death signaling. Based upon these results, we speculate that the intracellular oxidative stress, which is produced by genetically impaired mitochondrial ROS, might preferentially occur in astrocytes and accumulate as the antioxidant activity diminishes with age. In addition, Ca^2+^ overload serves as an oxidative stress marker in neuronal degenerative disease's pathophysiological phenotypes. For example, it has been reported that increasing Ca^2+^ influx and Ca^2+^ overload assist in promoting cell death with oxidative stress by ischemia–reperfusion (Kristian & Siesjo, 1998). In addition, toxic forms of β‐amyloid induce Ca^2+^ influx into neurons by inducing membrane‐associated oxidative stress or by forming oligomeric membrane pores, thereby rendering neurons vulnerable to excitotoxicity and apoptosis (Bezprozvanny & Mattson, 2008). Our results in middle‐aged *Tet‐mev‐1* mice suggest that Ca^2+^ overload could be an initial marker of pathophysiological changes. Therefore, the appearance of astrocyte defects with JNK/SAPK activation and Ca^2+^ overload might be an important age‐dependent physiological change in the central nervous system with impaired mitochondrial oxidative stress. These age‐dependent, oxidative stress‐sensitive physiological changes might disrupt the neuron‐astrocyte cross talk. For example, the exchange from glucose to pyruvate through lactate transport from astrocytes to neurons is required for activated neurons in memory consolidation (Suzuki *et al*., 2011). We demonstrated that the genetically impaired mitochondrial ROS production, which ultimately resulted in chronic physiologically relevant oxidative stress, decreased basal and inductive phosphorylated CREB levels independent of cAMP‐ and Ca^2+^/CaM‐dependent signal transduction in middle‐aged *Tet‐mev‐1* mice.

In general, it has been reported that a number of neurodegenerative diseases involve astrogliosis, which is clinically characterized as abnormal morphology and excessive proliferation of astrocytes (Sofroniew, 2009). However, astrocyte loss has also been implicated in Alzheimer's disease, FTD, and hypoxic–ischemic injury (Kim *et al*., 2011; Ryu *et al*., 2011a,b; Misu *et al*., 2013; Sakai *et al*., 2013). This includes autophagic astroglial death defined by cytoplasmic swelling and vacuolation, beading and dissolution of their processes (clasmatodendrosis), and nuclear alterations resembling apoptosis.

**Figure 7:**
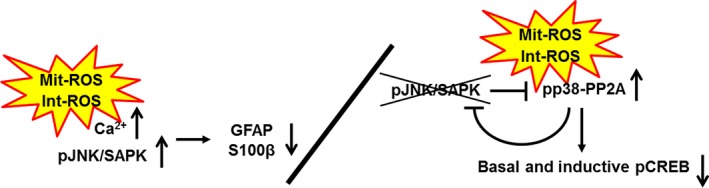
Graphical summary of the effects of chronic physiologically relevant oxidative stress from impaired mitochondria. The oxidative stress upregulates JNK/SAPK cell death signaling with Ca^2+^ overload in astrocytes. The resulting astrocyte defects could cause disrupted memory consolidation via diminished basal and inductive phosphorylated CREB with activating p38 MAPK‐PP2A signals. Alternatively, or in addition, memory consolidation could be impacted by the effects of oxidative stress directly on neurons.

We propose that the effects of chronic physiologically relevant oxidative stress can lead to astrocyte defects. In turn, these might cause the age‐dependent CREB inactivation in neurons via age‐dependent polarity changes of p38 MAPK‐PP2A signal transduction. Young adults are able to adapt to oxidative stress with inactivating p38 MAPK‐PP2A signals. In contrast, middle‐aged adults upregulate JNK/SAPK cell death signaling with Ca^2+^ overload in astrocytes. The resulting astrocyte defects could cause disrupted memory consolidation via diminished basal and inductive phosphorylated CREB with activating p38 MAPK‐PP2A signals. As illustrated in the graphical summary (Fig. 7), we could not specifically identify negative effects on neuron‐astrocyte cross talk. However, we demonstrated a number of molecular changes consistent with this, including JNK/SAPK activation in astrocytes, Ca^2+^ overload in astrocytes, CREB inactivation via activating p38 MAPK‐PP2A signals in neuronal activity. This could have resulted in the observed disrupted memory consolidation. In addition, the memory consolidation defects we observed could be partially attributed to direct neuronal damage by chronic oxidative stress. We anticipate that *Tet‐mev‐1* mice will continue to be a suitable model to clarify the effects of oxidative stress on neuron‐astrocyte cross talk as well as to study the effects of oxidative stress on specifically on neurons or astrocytes.

## Experimental procedures

### Animals

Both wild‐type C57BL/6J as controls and C57BL/6J background *Tet‐mev‐1* conditional transgenic mice were treated with doxycycline hyclate (0.1 mg mL^−1^ doxycycline with 0.01 mg mL^−1^ saccharine for fertile and pregnant mice or 0.4 mg mL^−1^ doxycycline with 0.05 mg mL^−1^ saccharine for experimental mice after weaning in drinking water) throughout prenatal development to adulthood. Young adult (4–8 months old) and middle‐aged (10–14 months old) mice were used for all studies. All animals were maintained on a 12‐h light/dark cycle under a specific‐pathogen free (SPF) condition: 22 ± 1 °C, approximately 40% humidity, 12/12‐h light/dark cycle, a standard diet CE2 (9.3% water, 25.8% crude protein, 4.5% crude fat, 4.5% crude fiber, 6.7% crude ash; CLEA Japan, Inc., Tokyo, Japan), and sterile water with doxycycline and saccharine *ad libitum*. All protocols complied with the Guidelines for Animal Experimentation of Tokai University and were approved by the Tokai University Animal Care Committees.

### Hippocampal tissue culture

The brains were removed and placed in ice‐cold Hank's solution (NISSUI Pharmaceutical Co., Tokyo, Japan), which was continuously bubbled with 95% O_2_ + 5% CO_2_ for over 30 min after deaeration. Coronal slices of 250 μm thickness were cut with a vibratome (LEICA VT 1000 S; Leica Microsystems, Tokyo, Japan) and transferred into a mesh‐bottom holding chamber containing Hank's solution bubbled with a mixture of 95% O_2_ + 5% CO_2_, leaving the final pH at 7.4. The slices were kept at 37 °C until the onset of the experiments.

### Histological chemical fluorometric analyses on *in vitro*‐cultured brain sections

Mitochondrial ROS production was monitored at 37 °C by using the oxidation‐sensitive red fluorescence dye MitoSOX Red (Molecular Probes: M36008), which is mitochondrially targeted probe and sensitive to mitochondrial ROS, mainly superoxide anion by the electron leakage from mitochondria. The dye (50 μg) was freshly dissolved each day in 13 μL dimethyl sulfoxide (DMSO). The slices were loaded with 5 μm MitoSOX Red including Hank's medium for 10 min at 37 °C, some of which were pretreated with 10 μL semiquinone‐like inhibitor HQNO, which inhibits electron transport (Van Ark & Berden, 1977; Christenson *et al*., 2008) (2 mg mL^−1^) for 30 min at 37 °C. HQNO treatment leads to electron leakage from the electron transport chain. This results in artificial mitochondrial ROS production (as detected with MitoSOX Red). After loading, the slices were washed three times and then left in Hank's solution.

Intracellular ROS production was monitored at 37 °C by using the oxidation‐sensitive dye 5‐(and‐6)‐chloromethyl‐2′,7′‐dichlorodihydrofluorescein diacetate, acetyl ester (CM‐H_2_DCFDA) which is sensitive to hydrogen peroxide (H_2_O_2_) and hydroxyl radical (HO˙; Molecular Probes: C6827). CM‐H_2_DCFDA is a nonfluorescent dye until the acetate groups are removed by intracellular esterases and oxidation occurs within the cell. The dye (50 μg) was freshly dissolved each day in 10 μL DMSO. The slices were loaded with 10 μm CM‐H_2_DCFDA in the dark (30 min; 37 °C; 0.02% [w/v] Pluronic F127). After loading, the slices were washed 5 times and then left in Hank's solution. Five millimolar probenecid was added to every solution used after loading to inhibit organic anion transporters that remove fluorescent dyes from the cytoplasm in a temperature‐dependent way.

Intracellular overloaded Ca^2+^ levels were monitored at 37 °C by using the rhodamine‐like fluorescent indicator Rhod‐2‐AM, 1‐[2‐Amino‐5‐(3‐dimethylamino‐6‐dimethylammonio‐9‐xanthenyl)phenoxy]‐2‐(2‐amino‐5‐methylphenoxy)ethane‐*N*,*N*,*N*′,*N*′‐tetraacetic acid, tetraacetoxymethyl ester, chloride (Dojindo: R002, 341‐05821). This indicator's dissociation constant (*K*_d_ **= 1.0 μmol L^−1^) is higher than the physiological range of intracellular Ca^2+^ concentration, <200 nmol L^−1^, and less than extracellular fluid Ca^2+^ concentration, about 1 mmol L^−1^ (Minta *et al*., 1989). The dye (50 μg) was freshly dissolved each day in 100 μL DMSO. The slices were loaded with 4.5 μm Rhod‐2 in the dark (30 min; 37 °C; 0.02% [w/v] Pluronic F127). After loading, the slices were washed five times and then left in Hank's solution.

Slices were submerged and mounted by a cover glass in 35‐mm tissue culture dishes, excited at 510 nm for MitoSOX Red, 488 ± 5 nm for CM‐H_2_DCFDA, or 553 nm for Rhod‐2 and viewed under the Biorevo BZ‐9000 fluorescence microscope (KEYENCE, Osaka, Japan). The emitted light (580 nm for MitoSOX Red, 535 ± 25 nm for CM‐H_2_DCFDA, or 567 nm for Rhod‐2) was detected and the system was controlled with the BZ Analyzer II software (KEYENCE, Osaka, Japan). The image frame rate and exposure time were held constant for all experiments. Three layers in the hippocampal region, CA1, CA3, and dentate gyrus (DG), were analyzed by a line profile quantification analysis of BZ Analyzer II software. Autofluorescence correction in each examined region of every individual hippocampal slice was made by point‐by‐point subtraction of the respective average intensity values of identical experiments performed on unloaded slices.

### Western blot analyses

Forty micrograms of total protein extract solubilized by boiling after the addition of 2 × SDS‐PAGE sample buffer [0.125 m Tris–HCl (pH 6.8), 10% 2‐mercaptoethanol, 4% SDS, 10% sucrose, and 0.004% bromophenol blue]/lane was resolved using 10–20% SDS‐PAGE gradient gel and analyzed by Western blot. After electrophoresis, the proteins were transferred to PVDF (polyvinylidene difluoride) Clear Blot P membrane (ATTO Corp, Tokyo, Japan) using a semi‐dry blotting machine AE‐6677 (ATTO Corp., Tokyo, Japan). To block nonspecific protein binding, membranes were treated for 2–8 h at 20–25 °C with 5% bovine serum albumin (#BAH64; Equitech‐Bio, Inc., Kerrville, TX, USA) and 0.1% Tween‐20 in TBS [0.02 m TRIZMA BASE, 0.137 m NaCl (pH 7.6)]. Primary antibodies phospho‐SAPK/JNK (Thr183/Tyr185) antibody (#9251S; Cell Signaling Technology, Japan, K.K., Tokyo, Japan), anti‐GFAP antibody (Z0334; Dako, Japan, Tokyo, Japan), anti‐tubulin beta‐3 antibody (NB100‐1612; Novus Biologicals, Littleton, CO, USA), phospho‐CREB (Ser133; 87G3) rabbit mAb, CREB (48H2) rabbit mAb antibodies, PP2A C subunit (52F8) rabbit mAb and phospho‐p38 MAP kinase (Thr180/Tyr182) antibody ( #9198, #9197, #2259P and #9211S; Cell Signaling Technology), anti‐phospho‐PKA catalytic β subunit (Ser338) and PKAβ cat (C‐20) antibodies (07‐868; Merck Millipore, Darmstadt, Germany and sc‐904; Santa Cruz Biotechnology, London, UK), p‐CaMKIV (Thr 196)‐R and CaMKIV antibodies (sc‐28443‐R; Santa Cruz Biotechnology and #4032; Cell Signaling Technology), phospho‐PP1α (Thr320) and PP1α antibodies (#2581S and #2582S; Cell Signaling Technology), pan‐calcineurin A antibody (#2614S; Cell Signaling Technology), mouse SDHC 23–59 peptide antibody was made by MBL company (Nagoya, Japan) that was previously reported (Ishii *et al*., 2011), rabbit monoclonal antibody [E115] to estrogen receptor alpha (GTX61047; GeneTex, Inc., Irvine, CA, USA), rabbit polyclonal estrogen receptor beta antibody (ab3576; Abcam), and anti‐beta actin antibody (GTX110564; GeneTex, Inc., Irvine, CA, USA) were used in TBS containing 5% bovine serum albumin in each condition. Actin was used as an internal control protein for loading normalization of the quantification analysis. Horseradish peroxidase‐coupled specific secondary antibodies, anti‐rabbit IgG, HRP‐linked antibody (#7074; Cell Signaling Technology), or polyclonal rabbit anti‐goat immunoglobulins/HRP antibody (P0449; Dako) were incubated in TBS containing 5% bovine serum albumin for 2 h at room temperature in each condition. The detection system used was ECL Plus Western Blotting Detection Reagent (GE Healthcare, UK). The chemiluminescence signals were visualized under LAS3000 mini (FUJIFILM Corp., Tokyo, Japan). Quantitative densitometric analysis was done using Multi Gauge Ver3.0 (FUJIFILM Corp., Tokyo, Japan).

### Immunohistochemistry

The segments of mouse brain were perfused and fixed with 4% paraformaldehyde in phosphate‐buffered saline (PBS) under physiological pressure and embedded in paraffin and sectioned at a thickness of 5 μm. Immunohistochemical analyses were performed using phospho‐SAPK/JNK (Thr183/Tyr185) antibody (#9251S; Cell Signaling Technology), anti‐GFAP antibody (Z0334; Dako), anti‐S100 beta antibody [EP1576Y] (ab52642; Abcam), and anti‐beta amyloid 1–42 antibody (ab14220; Abcam). The deparaffinized and dehydrated sections were incubated for 5 min at 120 °C in Dako Target Retrieval Solution (pH 9.0; S2367; Dako), treated with in 3% hydrogen peroxide in distilled water (dH_2_O), and incubated with phospho‐SAPK/JNK (Thr183/Tyr185) antibody diluted 1:100 in TBS [0.02 m TRIZMA BASE, 0.137 m NaCl (pH 7.6)], anti‐GFAP antibody (Z0334; Dako) diluted 1:500 in TBS, anti‐S100 beta antibody [EP1576Y] diluted 1:500 in TBS, and anti‐beta amyloid 1–42 antibody (ab14220; Abcam) diluted 1:500 in TBS in each reaction after blocking nonspecific protein binding in TBS containing 5% goat serum for 10 min. Sections were treated with Histofine^®^ Simple Stain^™^ Mouse MAX PO (R) (414341F; Nichirei Biosciences Inc., Tokyo, Japan) after washing in TBS and treated in diaminobenzidine and hematoxylin nuclear staining.

### Mitochondrial and cytosolic preparations

The mouse hippocampal area protein extracts were prepared by a Teflon homogenizer in a volume of isolation buffer [210 mm mannitol, 70 mm sucrose, 5 mm Tris–HCl (pH 7.4), and 0.1 mm EDTA] that was fivefold greater than the tissue weight. Mitochondria and cytosolic fraction proteins were isolated by differential centrifugation. The nuclear fraction and unbroken cells were first removed from total protein lysate of hippocampal area by centrifugating at 800 *g* for 30 min. The mitochondrial fraction of hippocampal area was then obtained by centrifugating at 21 000 *g* for 30 min. Finally, mitochondrial fraction protein was suspended in Tris–EDTA buffer [50 mm Tris–HCl (pH 7.4) and 5 mm EDTA].

### Biochemical materials

Biochemical analyses were performed using (i) a SOD Assay kit‐WST (S311; Dojindo Molecular Technologies, Inc., Kumamoto, Japan) for SOD activity measurements; (ii) 2‐methyl‐6‐*p*‐methoxyphenylethynyl‐imidazopyrazinone (MPEC) chemical luminescent probe (ATTO Corp, Tokyo, Japan) for the measurement of ROS level in submitochondrial particles *in vitro*; (iii) Lactate Pro^™^ with Lactate Pro^™^ Test Strip (Arkray, Inc., Kyoto, Japan) for the measurement of lactate level in the cytosolic fraction of hippocampal area; (iv) SensoLyte^R^ 520 Thiol Quantification Kit *Fluorimetric* (AnaSpec, Inc., Fremont, CA, USA) for the quantification analysis of total thiols level; and (v) GSSG/GSH Quantification Kit (Dojindo Molecular Technologies, Inc.) for the GSSG/GSH ratio quantification analysis.

### Morris water maze

Morris water maze testing was performed using young adult (4–8 months old) and middle‐aged (10–14 months old) female mice with the experimenter blind to genotype. Mice were handled for 5 min each for five consecutive days before beginning experiments. The arena consisted of a circular pool (diameter of 170 cm) filled with water that was temperature‐regulated (24–30 °C) and made opaque with nontoxic white tempera paint. A circle, plexiglass platform (diameter of 15 cm) was submerged 1 cm below the surface of the water and four local cues were provided to allow spatial map generation. Mice were trained swimming and escaping on the platform for 1–2 weeks. Learning occurred at a total of 15 trials over 5 days, with one session every 2 h three times per day or a total of six trials over 2 days, with one session every 2 h per day more than 1 week later after last training trial. Start locations (four local cues for the spatial map) were pseudo‐randomized so that each start location was used once per session and the sequence of start locations in any session was never used twice. Tracking and analysis of animal movement was done using the SMART Video Tracking System Version 2.5 (Panlab, Barcelona, Spain).

### Data analyses

One‐way anova and Bonferroni *post hoc* comparisons were used to analyze the difference between probe trials in the Morris water maze for pairwise comparisons. Planned comparisons, using a two‐tailed, unpaired Student's *t*‐test, were used to analyze differences of the time spent in the target quadrant (TQ) in the Morris water maze between two groups. Statistically, measurements of biochemical assay and Western blot were analyzed by one‐way anova, Bonferroni *post hoc* comparisons, and a two‐tailed, unpaired Student's *t*‐test. All values in the figures and figure legends are means ± SD.

## Author contributions

Takamasa Ishii was involved in the conception and design, collection and assembly of data, data analysis and interpretation, manuscript writing, and final approval of manuscript. Yumi Takanashi contributed to assembly of data, data analysis and interpretation, and final approval of the manuscript. Masaki Miyazawa contributed to data collection and analysis and approved the manuscript. Hiromi Onouchi, Noboru Kawabe, Munehiro Nakata and Yorihiro Yamamoto contributed to data collection and approved the manuscript. Phil S. Hartman contributed to conception and design, data analysis and interpretation, manuscript writing, and final approval of manuscript. Naoaki Ishii contributed to conception and design, financial support, administrative support, data analysis and interpretation, manuscript writing, and final approval of manuscript.

## Conflict of interest

The authors have no conflict of interest directly relevant to the content of this article.

## Funding

This work was supported by the KAKENHI (22689012) in Grant‐in‐Aid for Young Scientists (A) and KAKENHI (23650602) in Grant‐in‐Aid for Challenging Exploratory Research from the Ministry of Education, Culture, Sports, Science and Technology (MEXT) and Japan Society for the Promotion of Science (JSPS), and the Tokai University School of Medicine Research Aid.

## Supporting information


**Fig. S1** *In vitro* biochemical assays in doxycycline‐treated mice.
**Fig. S2** β‐Amyloid 1–42 accumulation levels in the young adult (4–8 months old; YA), middle‐aged (10–14 months old; MA) and senior‐aged (24–28 months old; SA) wild‐type C57BL/6J and *Tet‐mev‐1* mice.
**Fig. S3** TUNEL positive cells in the young adult (4–8 months old; YA), middle‐aged (10–14 months old; MA) and senior‐aged (24–28 months old; SA) wild‐type C57BL/6J and *Tet‐mev‐1* mice.
**Fig. S4** Lactate levels in the young adult (4–8 months old; YA), middle‐aged (10–14 months old; MA) and senior‐aged (24–28 months old; SA) mice.
**Fig. S5** High magnification images of strong fluorescence in middle‐aged (10–14 months old) *Tet‐mev‐1* mice.
**Fig. S6** Total thiols, glutathione (GSH) and glutathione disulfide (GSSG) levels in young adult (4–8 months old; YA) and middle‐aged (10–14 months old; MA) mice.
**Fig. S7** Carbonylated protein ratios in young adult (4–8 months old; YA) and middle‐aged (10–14 months old; MA) mice.
**Fig. S8** High magnification images of stress‐activated protein kinases (SAPK)/c‐Jun N‐terminal kinases (JNK)‐activated and Ca^2+^‐overloaded cells of middle‐aged (10–14 months old) *Tet‐mev‐1* mice.
**Fig. S9** Swimming velocities and distance traveled in a Morris‐Water Maze.
**Fig. S10** Spatial learning abilities in a Morris‐water maze.
**Fig. S11** The activities of cAMP‐ and Ca^2+^/CaM‐dependent protein kinases and Ser/Thr protein phosphatases in the young adult (4–8 months old; YA) and middle‐aged (10–14 months old; MA) wild‐type C57BL/6J and *Tet‐mev‐1* mice.
