# Supplementary material for: Endogenous reactive oxygen species cause astrocyte defects and neuronal dysfunctions in the hippocampus: a new model for aging brain
Source: Aging Cell. 2016 Sep 13;16(1):39–51. doi: 10.1111/acel.12523 (PMC5242301; doi:10.1111/acel.12523)
Supplement: Supplementary file 1 — Fig. S1 In vitro biochemical assays in doxycycline‐treated mice. Fig. S2 β‐Amyloid 1–42 accumulation levels in the young adult (4–8 months old; YA), middle‐aged (10–14 months old; MA) and senior‐aged (24–28 months old; SA) wild‐type C57BL/6J and Tet‐mev‐1 mice. Fig. S3 TUNEL positive cells in the young adult (4–8 months old; YA), middle‐aged (10–14 months old; MA) and senior‐aged (24–28 months old; SA) wild‐type C57BL/6J and Tet‐mev‐1 mice. Fig. S4 Lactate levels in the young adult (4–8 months old; YA), middle‐aged (10–14 months old; MA) and senior‐aged (24–28 months old; SA) mice. Fig. S5 High magnification images of strong fluorescence in middle‐aged (10–14 months old) Tet‐mev‐1 mice. Fig. S6 Total thiols, glutathione (GSH) and glutathione disulfide (GSSG) levels in young adult (4–8 months old; YA) and middle‐aged (10–14 months old; MA) mice. Fig. S7 Carbonylated protein ratios in young adult (4–8 months old; YA) and middle‐aged (10–14 months old; MA) mice. Fig. S8 High magnification images of stress‐activated protein kinases (SAPK)/c‐Jun N‐terminal kinases (JNK)‐activated and Ca2+‐overloaded cells of middle‐aged (10–14 months old) Tet‐mev‐1 mice. Fig. S9 Swimming velocities and distance traveled in a Morris‐Water Maze. Fig. S10 Spatial learning abilities in a Morris‐water maze. Fig. S11 The activities of cAMP‐ and Ca2+/CaM‐dependent protein kinases and Ser/Thr protein phosphatases in the young adult (4–8 months old; YA) and middle‐aged (10–14 months old; MA) wild‐type C57BL/6J and Tet‐mev‐1 mice. [file ACEL-16-39-s001.docx]

**
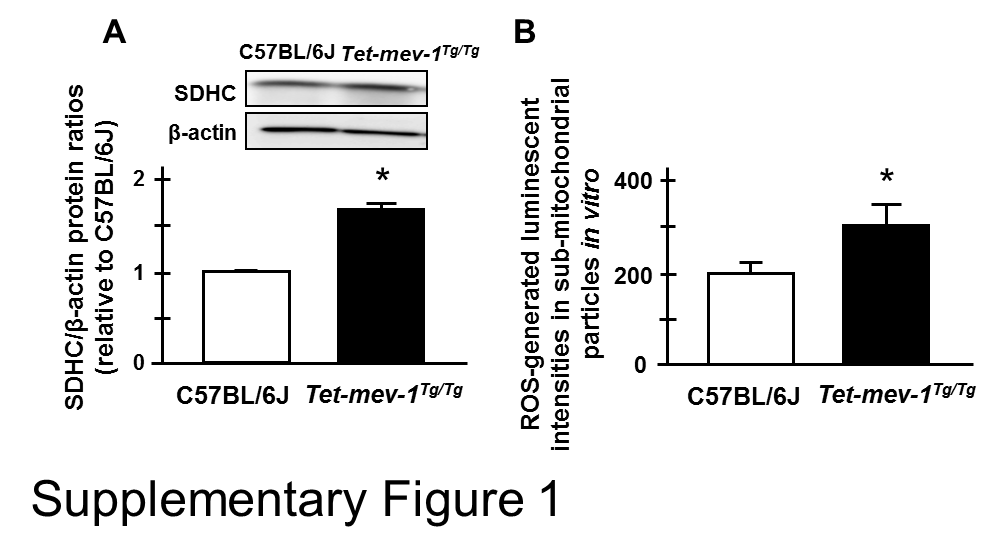
**

**Fig. S1.** *In vitro* biochemical assays in doxycycline-treated mice. (*A*) SDHC/β-actin protein levels by western blot analysis. White- and black-bars indicate the wild-type C57BL/6J and *Tet-mev-1* mice, respectively. Data are expressed as mean ± SD; **P* < 0.01; *N* = 3 in each group. (*B*) Reactive oxygen species (ROS)-generated luminescent intensities in sub-mitochondrial particles using 2-methyl-6-*p*-methoxyphenylethynyl-imidazopyrazinone) luminescent chemical probe (MPEC) (ATTO Corporation). Data are expressed as mean ± SD; **P* < 0.01; *N* = 3 in each group.

**
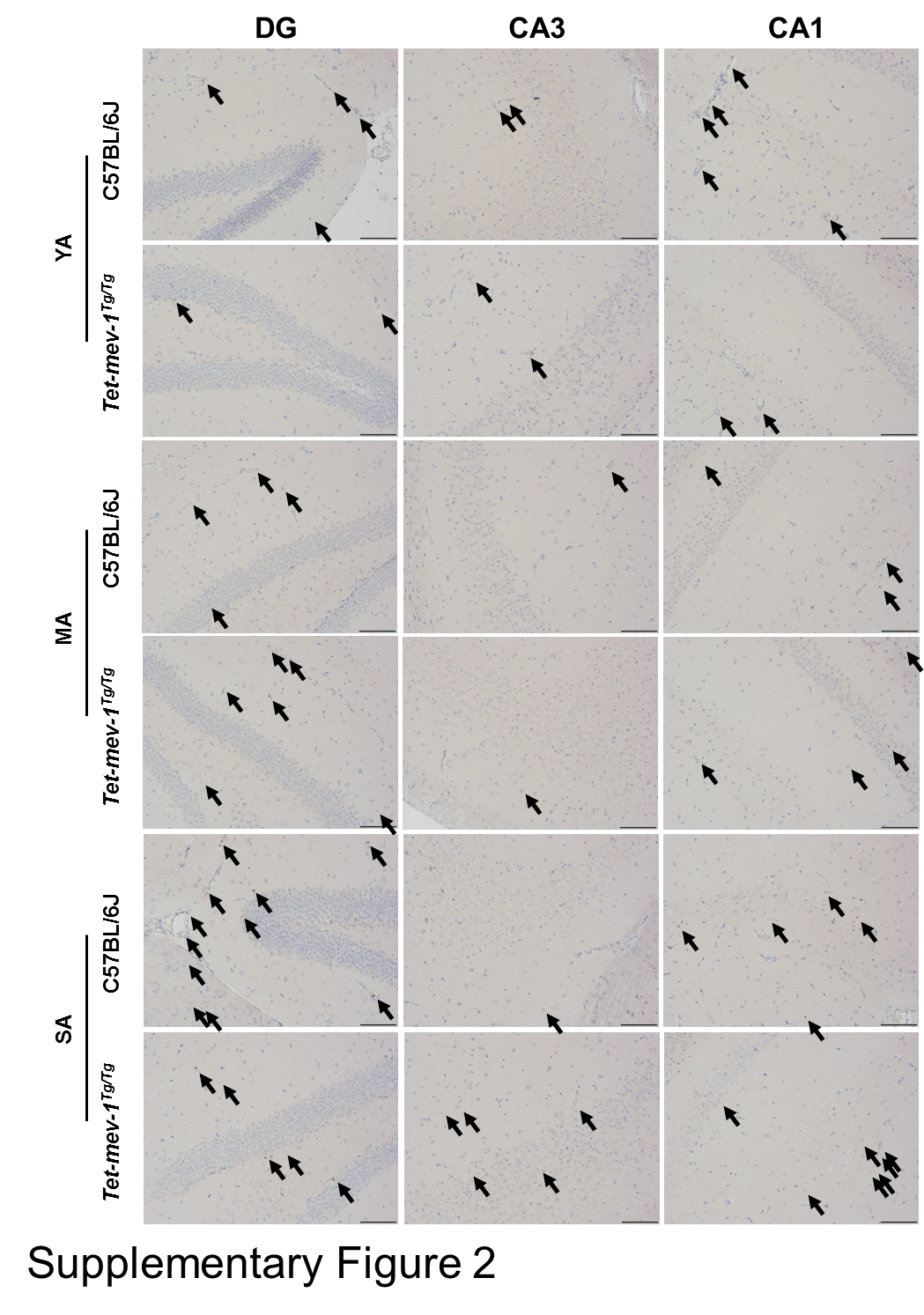
**

**Fig. S2.** β-amyloid 1-42 accumulation levels in the young adult (4-8 months old; YA), middle-aged (10-14 months old; MA) and senior-aged (24-28 months old; SA) wild-type C57BL/6J and *Tet-mev-1* mice. β-amyloid 1-42 accumulation levels by immunohistochemical analysis using Anti-beta Amyloid 1-42 antibody (Abcam: ab10148) to whole brain paraffin sections. Block allows indicate β-amyloid 1-42-accumulated brown color cells. Scale bar = 100µm.

**
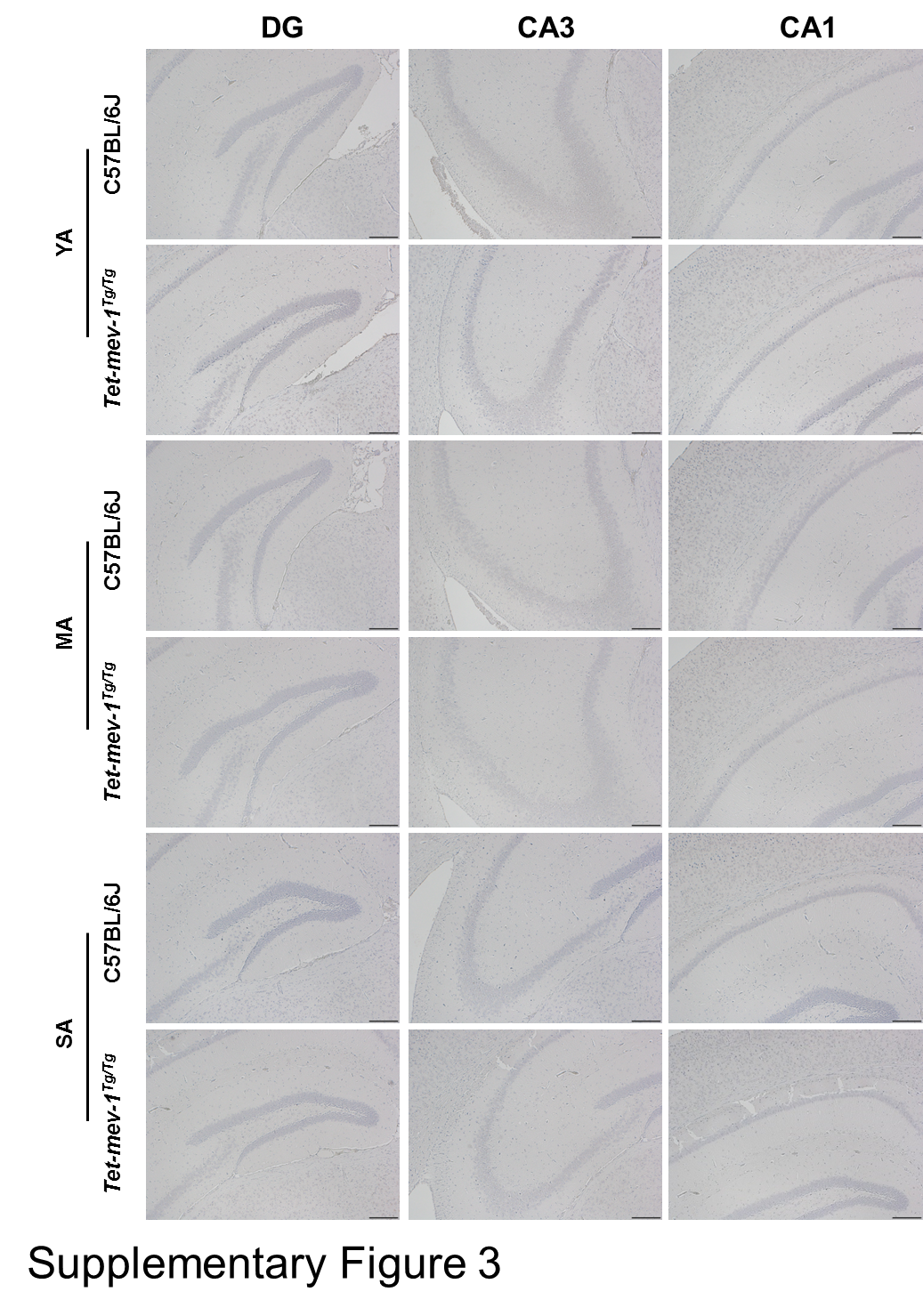
**

**Fig. S3.** TUNEL positive cells in the young adult (4-8 months old; YA), middle-aged (10-14 months old; MA) and senior-aged (24-28 months old; SA) wild-type C57BL/6J and *Tet-mev-1* mice. TUNEL staining on whole brain paraffin sections using TUNEL Enzyme, TUNEL Dilution Buffer and Biotin-16-dUTP according to the manufacturer’s instructions (Roche Diagnostics Corp., Tokyo, Japan) that was previously reported (Ishii et al. 2011). Brown cells indicate the TUNEL-positive cells, meaning apoptotic cells. Scale bar = 100µm.

**
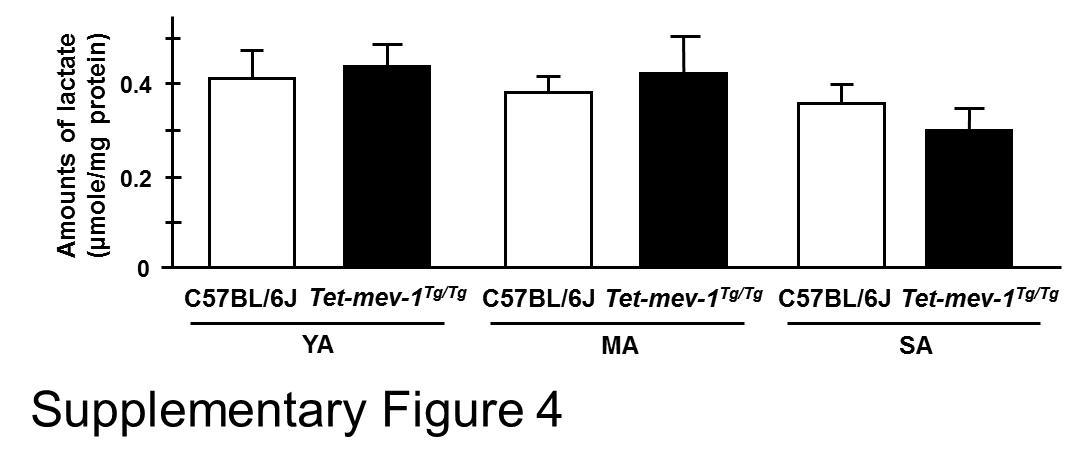
**

**Fig. S4.** Lactate levels in the young adult (4-8 months old; YA), middle-aged (10-14 months old; MA) and senior-aged (24-28 months old; SA) mice. Lactate levels in cytosolic fractions of hippocampal area were measured by Lactate Pro^TM^ with Lactate Pro^TM^ Test Strip (ARKRAY, Inc.). White and black bars indicate the wild-type C57BL/6J and *Tet-mev-1* mice, respectively. Data are expressed as mean ± SD; *N* = 8 in each group.


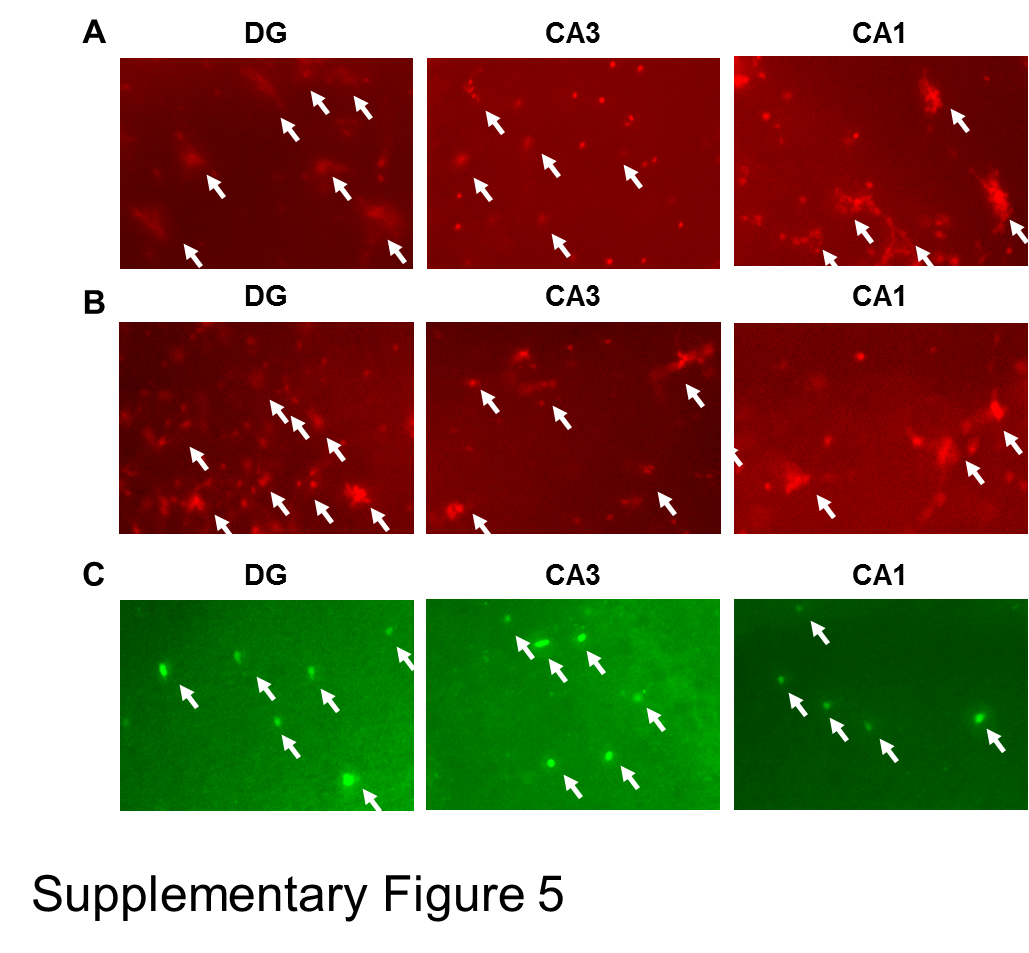


**Fig. S5.** High magnification images of strong fluorescence in middle-aged (10-14 months old) *Tet-mev-1* mice. (*A*) The high magnification micrographs of mitochondrial ROS-generated red-fluorescence in the dentate gyrus (DG), CA3 and CA1 regions in figure 1*A*. White allows indicate the strong fluorescent-positive cells with large cell body like astrocytes in the other areas, not granular and pyramidal layers. (*B*) The high magnification micrographs of mitochondrial ROS-generated red-fluorescence in the dentate gyrus (DG), CA3 and CA1 regions in figure 2*A*. White allows indicate the strong fluorescent-positive cells with large cell body like astrocytes in the other areas, not granular and pyramidal layers. (*C*) The high magnification micrographs of intracellular ROS-generated green-fluorescence in the dentate gyrus (DG), CA3 and CA1 regions in figure 3*B*. White allows indicate the strong fluorescent-positive particle aggregates in the other areas, not granular and pyramidal layers.


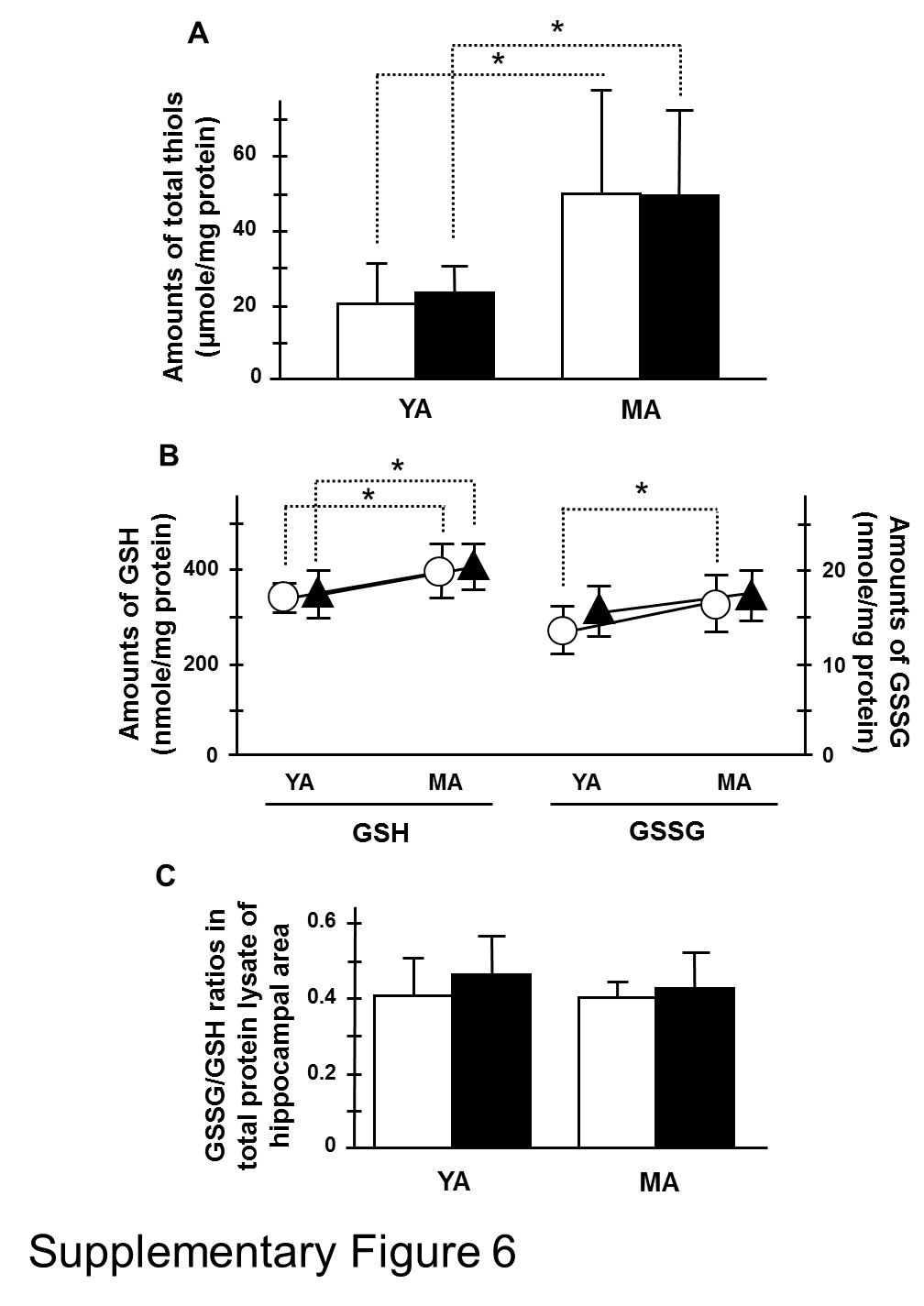


**Fig. S6.** Total thiols, glutathione (GSH) and glutathione disulfide (GSSG) levels in young adult (4-8 months old; YA) and middle-aged (10-14 months old; MA) mice. (*A*) The amount of total thiols in cellular extracts including protein lysate of hippocampal area. White- and black-bars indicate the wild-type C57BL/6J and *Tet-mev-1* mice, respectively. (*B*) GSH and GSSG in total cellular extracts of hippocampal area. White-circles and black-triangles indicate the wild-type C57BL/6J and *Tet-mev-1* mice, respectively. (*C*) The GSSG/GSH ratio in total cellular extracts of hippocampal area. White- and black-bars indicate the wild-type C57BL/6J and *Tet-mev-1* mice, respectively. All data are expressed as mean ± SD; **P* < 0.05; *N* = 8 in each group.


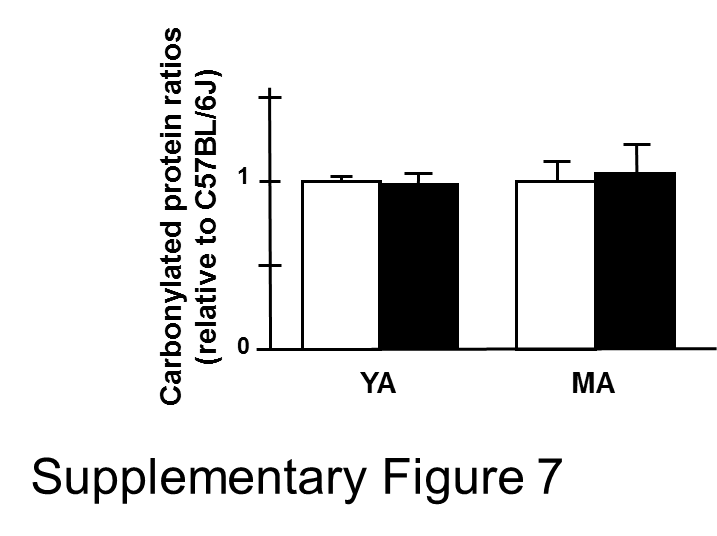


**Fig. S7.** Carbonylated protein ratios in young adult (4-8 months old; YA) and middle-aged (10-14 months old; MA) mice. Carbonylated protein is well known as an oxidative damaged protein marker in the cellular components. Carbonylated protein levels were measured after DNPH treatment of 200 ng total protein. The protein carbonyls content was quantified by enzyme-linked immunosorbent assay (ELISA) using 96-well plate (Corning Coster, Cambridge, MA) as previously reported (Ishii et al. 2014). White and black bars indicate the wild-type C57BL/6J and *Tet-mev-1* mice, respectively. Data are expressed as mean ± SD; *N* = 3 in each group.


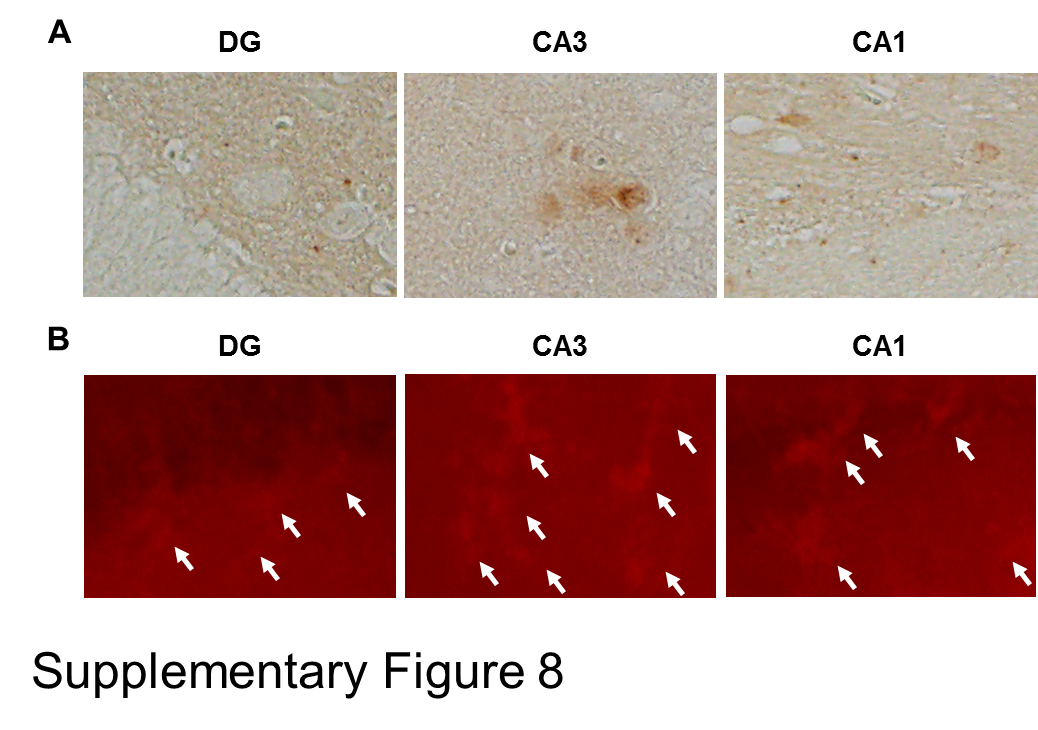


**Fig. S8.** High magnification images of stress-activated protein kinases (SAPK)/c-Jun N-terminal kinases (JNK)-activated and Ca^2+^-overloaded cells of middle-aged (10-14 months old) *Tet-mev-1* mice. (*A*) The high magnification micrographs of immunohistochemistry using phospho-SAPK/JNK (Thr183/Tyr185) antibody in the dentate gyrus (DG), CA3 and CA1 regions in figure 4*C*. Brown images indicate the immunostaining positive cells in the other areas, not granular and pyramidal layers. (*B*) The high magnification micrographs of Ca^2+^-overloaded red-fluorescent cells with Rhod 2-AM in the dentate gyrus (DG), CA3 and CA1 regions in figure 4*D*. White arrows indicate Ca^2+^-overload red-fluorescent positive cells with large cell body like astrocytes in the other areas, not granular and pyramidal layers.


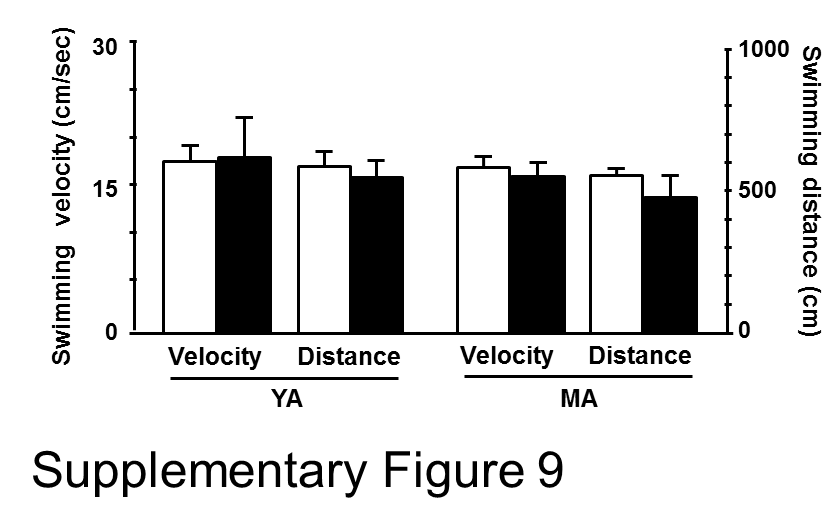


**Fig. S9.** Swimming velocities and distance traveled in a Morris-Water Maze. Swimming velocities and distances are showed by white- and black-bars indicating the young adult (4-8 months old; YA) and middle-aged (10-14 months old; MA) wild-type C57BL/6J and *Tet-mev-1* mice, respectively. Data are expressed as mean ± SD; *N* = 9 or 6 in each group of middle-aged C57BL/6J or *Tet-mev-1* mice, *N* = 12 or 11 in each group of young adult C57BL/6J or *Tet-mev-1* mice.

**
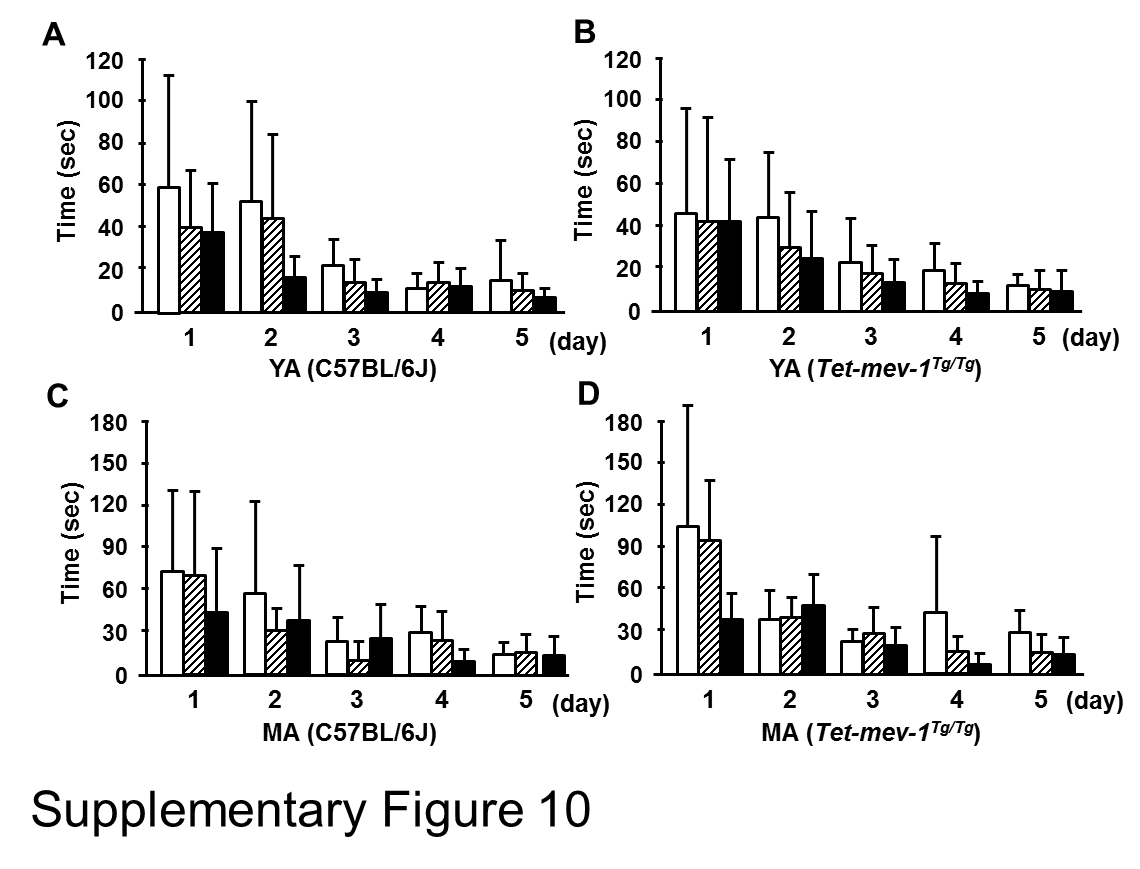
**

**Fig. S10.** Spatial learning abilities in a Morris-water maze. (*A***-***D*) Learning abilities in training using hidden platform trials in a Morris-water maze. Learning abilities are showed by white, diagonal and black bars indicate the first, second and third trials for the learning periods in the young adult (4-8 months old; YA, *A* and *B*) and middle-aged (10-14 months old; MA, *C* and *D*) of wild-type C57BL/6J (*A* and *C*) and *Tet-mev-1* mice (*B* and *D*), respectively. Data are expressed as mean ± SD; *N* = 8 or 10 in each group of young adult C57BL/6J or *Tet-mev-1* mice, *N* = 9 or 6 in each group of middle-aged C57BL/6J or *Tet-mev-1* mice.

**
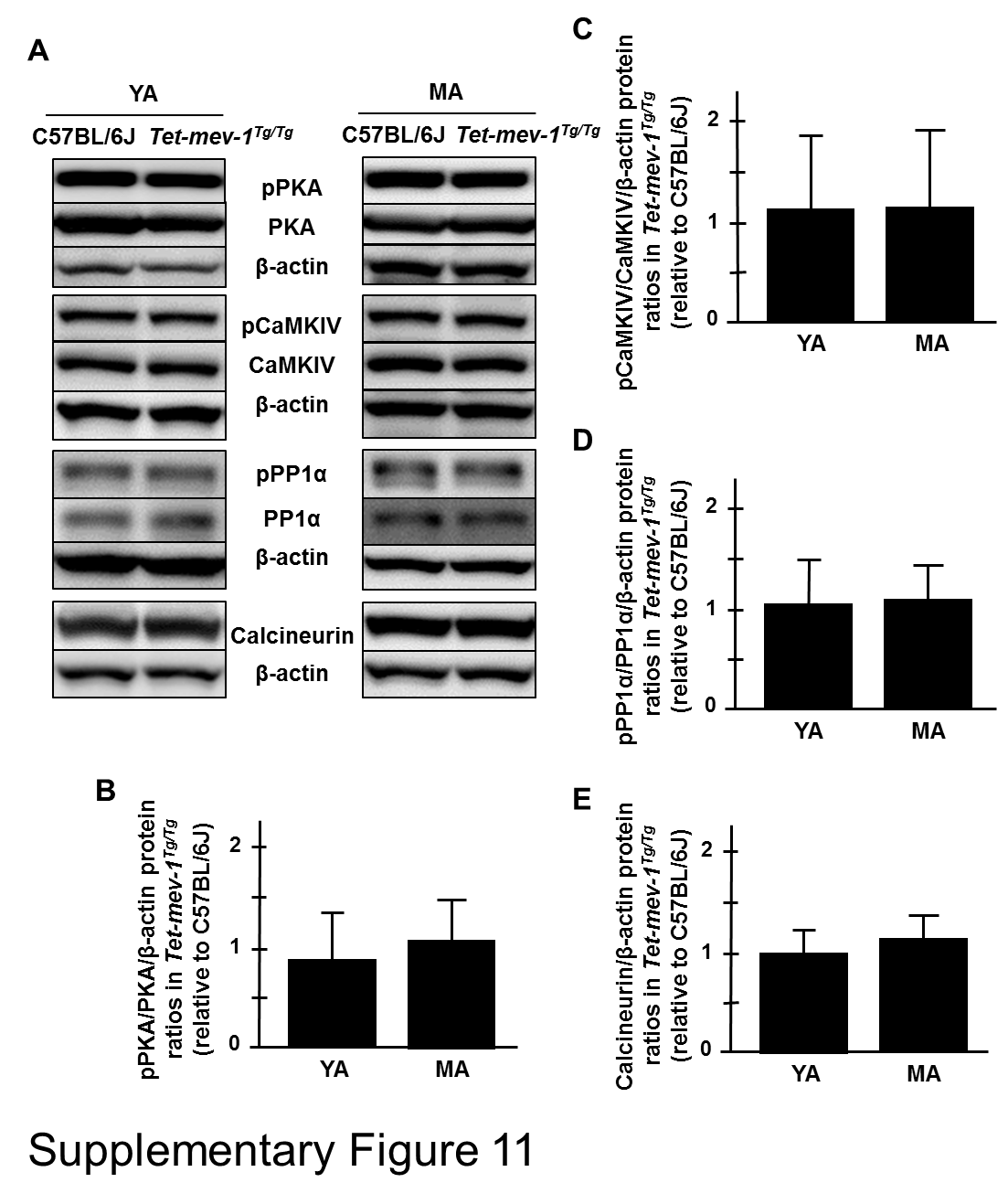
**

**Fig. S11.** The activities of cAMP- and Ca^2+^/CaM-dependent protein kinases and Ser/Thr protein phosphatases in the young adult (4-8 months old; YA) and middle-aged (10-14 months old; MA) wild-type C57BL/6J and *Tet-mev-1* mice. (*A*) The western blot images using anti-phospho-PKA catalytic β subunit (Ser338) and PKAβ cat (C-20) antibodies (Merck Millipore: 07-868 and SANTA CRUZ BIOTECHNOLOGY: sc-904), p-CaMKIV (Thr 196)-R and CaMKIV antibodies (SANTA CRUZ BIOTECHNOLOGY: sc-28443-R and Cell Signaling Technology: #4032), phosphor-PP1α (Thr320) and PP1α antibodies (Cell Signaling Technology: #2581S and #2582S), pan-calcineurin A antibody (Cell Signaling Technology: #2614S), and Rabbit polyclonal antibody to Beta-Actin (GeneTex: GTX110564)] to hippocampal protein lysate of young adult and middle-aged wild-type C57BL/6J and *Tet-mev-1* mice. (*B*-*E*) The internal standardized ratios of phosphorylated PKA/PKA (*B*). phosphorylated CaMKIV/CaMKIV (*C*), phosphorylated PP1/PP1(*D*), Calmodulin-binding catalytic subunit of calcineurin, calcineurin A level (*E*) by β-actin levels relative to young adult wild-type C57BL/6J. All data are expressed as mean ± SD; *N* = 5 in each age.
